# Peptide-biphenyl hybrid-capped AuNPs: stability and biocompatibility under cell culture conditions

**DOI:** 10.1186/1556-276X-8-315

**Published:** 2013-07-06

**Authors:** Mona Connolly, Yolanda Pérez, Enrique Mann, Bernardo Herradón, María L Fernández-Cruz, José M Navas

**Affiliations:** 1 Departamento de Medio Ambiente, Instituto Nacional de Investigación y Tecnología Agraria y Alimentaria (INIA), Carretera de la Coruña Km 7.5, Madrid 28040, Spain; 2 Universidad Rey Juan Carlos, Tulipán s/n, Móstoles, Madrid 28933, Spain; 3 Consejo Superior de Investigaciones Científicas (CSIC), Instituto de Química Orgánica General, Juan de la Cierva 3, Madrid 28006, Spain

**Keywords:** Gold nanoparticles, Hep G2, ROS, Autophagy, Cytotoxicity

## Abstract

In this study, we explored the biocompatibility of Au nanoparticles (NPs) capped with peptide-biphenyl hybrid (PBH) ligands containing glycine (Gly), cysteine (Cys), tyrosine (Tyr), tryptophan (Trp) and methionine (Met) amino acids in the human hepatocellular carcinoma cell line Hep G2. Five AuNPs, Au[(Gly-Tyr-Met)_2_B], Au[(Gly-Trp-Met)_2_B], Au[(Met)_2_B], Au[(Gly-Tyr-TrCys)_2_B] and Au[(TrCys)_2_B], were synthesised. Physico-chemical and cytotoxic properties were thoroughly studied. Transmission electron micrographs showed isolated near-spherical nanoparticles with diameters of 1.5, 1.6, 2.3, 1.8 and 2.3 nm, respectively. Dynamic light scattering evidenced the high stability of suspensions in Milli-Q water and culture medium, particularly when supplemented with serum, showing in all cases a tendency to form agglomerates with diameters approximately 200 nm. In the cytotoxicity studies, interference caused by AuNPs with some typical cytotoxicity assays was demonstrated; thus, only data obtained from the resazurin based assay were used. After 48-h incubation, only concentrations ≥50 μg/ml exhibited cytotoxicity. Such doses were also responsible for an increase in reactive oxygen species (ROS). Some differences were observed among the studied NPs. Of particular importance is the AuNPs capped with the PBH ligand (Gly-Tyr-TrCys)_2_B showing remarkable stability in culture medium, even in the absence of serum. Moreover, these AuNPs have unique biological effects on Hep G2 cells while showing low toxicity. The production of ROS along with supporting optical microscopy images suggests cellular interaction/uptake of these particular AuNPs. Future research efforts should further test this hypothesis, as such interaction/uptake is highly relevant in drug delivery systems.

## Background

At the forefront of many lines of research in drug delivery are the endless possibilities of gold nanoparticles (AuNPs) [1-4]. These molecules are readily taken up by cells, and they therefore provide a valuable means for drug delivery, with reports of efficient transport across the blood–brain barrier in mice [5] and nuclear penetration in the human HeLa cell line [6]. At nanoscale, the properties conferred upon such an otherwise inert metal in its bulk form are surprising. It is precisely these unique properties that offer potential in fields as diverse as diagnostics, anti-cancer therapies, catalysts and fuel cells. One avenue that has been studied exhaustively in recent years is the use of coatings and capping agents in the rational design of NPs, both to stabilise and functionalise these nanoparticles. Specific capping agents can lead to the self-assembly of NPs into ordered ‘superstructures’ creating different shapes [7], and by altering the capping structure, different arrangements can be achieved. In terms of biocompatibility, when using a polyvinyl alcohol capping agent, AuNPs do not show toxicity in zebrafish, despite being taken up into embryos and evidence of bioaccumulation [8]. These observations highlight the use of capping agents as an approach to achieve safer NPs. We recently proposed the use of peptide-biphenyl hybrid (PBH) ligands as capping agents [9]. PBHs have a biphenyl system and two amino acid/peptide fragments, and they present key characteristics, such as dynamic properties in solution [10], ordered structures in the solid phase [11] and biological activity as calpain inhibitors [12]. Some of these properties arise from the presence of amino acid residues, as well as aromatic rings, that are able to participate in a variety of non-covalent bonds, including hydrogen bonds [13,14] and arene interactions [15,16]. In addition, the conformational flexibility around the aryl-aryl single bond allows the PBH to adopt its structure in order to obtain the most favourable interactions with other chemical species, thus achieving high biological activity [17]. In peptidomimetics, this approach is considered a novel way to tailor NPs to have desired physico-chemical properties, which could contribute, for example, to advances in biomedical applications for AuNPs as drug delivery systems. A molecule can be designed in such a way as to benefit from structure-activity relationships and to attain higher levels of stability and/or biocompatibility. In a study on the design of peptide capping ligands for AuNPs, Lévy et al. [18] reported that peptide chain length, hydrophobicity and charge strongly influence NP stability. Here, we capped AuNPs with various PBH ligands and studied how the ligand structures influence the stability and the physico-chemical properties of the AuNPs under cell culture conditions and how they affect the biological response.

The huge discrepancies in reports on NP toxicity may be related to their different stabilities under various experimental conditions, leading to distinct physico-chemical properties that directly influence the effect of these particles. Given the unique and unpredictable behaviour of NPs in different environments [19,20], we performed a detailed physico-chemical analysis, a prerequisite for any NP toxicity study. Distinct NP properties, such as size, shape, aggregation state, zeta potential and dispersibility, along with the inherent composition of the NPs themselves, all influence the degree of toxicity [21-23]. To study the interaction between these PBH-capped AuNPs and biological systems, we undertook cytotoxicity studies. Many articles have demonstrated a close relationship between size and toxicity for AuNPs [24,25]. Findings suggest that size not only can influence uptake but may also dictate the possible interaction with DNA grooves [26,27], thus leading to AuNPs of different sizes showing distinct mechanisms of toxicity. For instance, AuNPs of 1.4 and 1.2 nm in diameter, thus differing by only 0.2 nm, show different pathways of toxicity in HeLa human cervix carcinoma cell lines, causing cell death by necrosis and apoptosis, respectively [28]. AuNPs have reported LC_50_ values of 65 to 75 μg/ml in *Daphnia magna*[29]. According to Farkas et al. [30], these particles are potent inducers of reactive oxygen species (ROS) in rainbow trout hepatocytes, with concentrations of 17.4 μg/ml increasing ROS production threefold as early as 2 h post-exposure. However, there have also been reports of AuNP biocompatibility, suggesting cell-selective responses following AuNP exposure that may be related to specific mechanisms of toxicity. Cell death through apoptosis has been reported in the human lung carcinoma cell line A549 after exposure to AuNPs, with no evidence of cytotoxicity in BHK21 (baby hamster kidney), Hep G2 (human hepatocellular liver carcinoma) or MDCK (canine epithelial kidney) cell lines [31,32]. These observations may be explained by AuNP interaction with cellular stress response mechanisms on a genetic level [33], which may dictate the cells capacity to prevent cytotoxic effects.

To further our understanding of AuNP interaction with biological systems and the properties that may govern biocompatibility, after performing a detailed physico-chemical characterisation of all the PBH-capped AuNPs, we used an *in vitro* approach to assess the possible toxic effects and the oxidative stress potential of these particles. We focused on how the structure of the capping PBH used affects NP size and stability over time under a range of conditions *in vitro*. Differences in NP behaviour when suspended in cell culture medium with serum and without serum were examined. This approach allowed us to compare any changes in the physico-chemical properties of the NPs that may be associated with the interaction of the agent with fetal bovine serum and protein coating. Given that the liver is one of the main sites of AuNP bioaccumulation following intravenous injection [34,35]; we chose a human hepatocellular carcinoma cell line (Hep G2) as the most appropriate and relevant test system. NPs have been described to interfere with assays, and some reviews report the limitations of certain assay systems [36] and that AuNPs even have the capacity to quench or enhance fluorescence depending on the plasmon field and dipole energy [37]. Also, gold can bind biological thiols such as glutathione [38,39]. Therefore, in this study, close attention was paid to any potential interference of AuNPs with the assay systems.

## Methods

### Chemicals and reagents

The synthesis and characterisation of PBHs are described in detail in Additional file 1. The chemicals used for AuNP synthesis, such as hydrogen tetrachloroaurate (III) trihydrate (HAuCl_4_∙3H_2_O), sodium borohydride (NaBH_4_), ethanol, 2-propanol and dimethyl sulfoxide-*d*_*6*_ were purchased from Sigma-Aldrich (Madrid, Spain).

For biocompatibility studies, Eagle’s minimum essential medium (EMEM), ultra glutamine 1 (200 mM in 0.85% NaCl solution), non-essential amino acids 100 X (NEAA), fetal bovine serum (FBS), penicillin/streptomycin (10,000 U/ml/10 mg/ml) and trypsin EDTA (200 mg/l EDTA, 17,000 U trypsin/l) were all sourced from LONZA (Barcelona, Spain). MEM and EMEM without phenol red were purchased from PAN Biotech GmbH (Aidenbach, Germany). High-grade purity water (>18 MΩ cm) obtained from a Milli-Q Element A10 Century (Millipore Iberia, Madrid, Spain) was used in all the experiments. All other chemicals were purchased from Sigma-Aldrich.

### Synthesis of AuNPs

**Figure 1:**
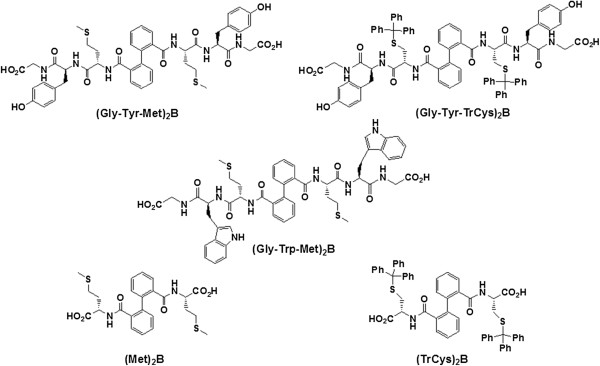
Peptide-biphenyl hybrid (PBH) ligands used in this study, Tr = Trityl, B = 2, 2’-(bis)carbonylbiphenyl.

Five AuNPs, (Au[(Gly-Trp-Met)_2_B], Au[(Gly-Tyr-TrCys)_2_B], Au[(Gly-Tyr-Met)_2_B], Au[(Met)_2_B] and Au[(TrCys)_2_B]) (Figure 1), were synthesised following the methodology described by Pérez et al. [9] (see Additional file 1). Thus, each PBH (50 μmol) was dissolved in ethanol (20 ml, 2.5 mmol/l) and was added to a solution of HAuCl_4_ (50 ml, 0.5 mmol/l) in 2-propanol under stirring. After 30 min, a freshly prepared aqueous solution of NaBH_4_ (4 ml, 50 mmol/l) was added slowly. The mixture was stirred for 2 h at room temperature to afford a red-brown colloidal gold solution. The AuNPs were precipitated by centrifugation for 15 min at 6,000 rpm. The black-brown precipitate was washed with 2-propanol to remove the free ligand and then dried under vacuum. The PBH-capped AuNPs obtained were stable to some cycles of precipitation and re-dispersion and could be easily dispersed in water.

### Physico-chemical characterisation of AuNPs

#### Transmission electron microscopy

Transmission electron microscopy (TEM) images of the synthesised AuNPs were obtained using a Philips Tecnai 20 operating at 200 kV (FEI, Eindhoven, The Netherlands). AuNPs were also examined after their suspension in culture medium without serum (EMEM/S-) at time 0 and 24 h using a LEO-910 microscope (Carl Zeiss, Oberkochen, Germany) operating at an accelerating voltage of 80 kV and equipped with a digital camera Gatan Bioscan 792 (Gatan Inc., Pleasanton, CA, USA). The samples for TEM characterisation were prepared by placing and evaporating a drop of the AuNPs in 2-propanol, or in medium, on carbon-coated copper grids (200 mesh). Average particle sizes were obtained by measuring the diameters of 150 particles.

#### Nuclear magnetic resonance

^1^H nuclear magnetic resonance (NMR) and ^13^C NMR spectra were recorded on Varian Mercury-400 and Varian Inova-300 instruments (Agilent Tecnologies, Santa Clara, CA, USA). Chemical shift (*δ*) constants are indicated in hertz. ^1^H NMR spectra were referenced to the chemical shift of TMS (*δ* = 0.00 ppm). ^13^C NMR spectra were referenced to the chemical shift of the deuterated solvent. The following abbreviations are used to explain multiplicities: s = singlet, d = doublet, t = triplet, q = quartet, m = multiplet, br = broad. The spectra of the ligands and the AuNPs were collected in dimethyl sulfoxide-*d*_6_ (DMSO-*d*_6_).

#### Elemental analysis

The amount of PBH capped on the AuNPs was estimated by elemental analysis of C, H, N and S. Combustion analyses were performed on an EA 1180-Elemental Analyzer (Carlo Erba, Milan, Italy).

#### Fourier transform infrared spectroscopy

Fourier transform infrared (FT-IR) spectra in the range of 600 to 4,000 cm^−1^ were recorded using a Nicolet-550 FT-IR spectrophotometer (Thermo Fisher, Hudson, NH, USA). The analysis was done in the solid state. Thirty-two scans were used to record the IR spectra.

#### UV–vis spectroscopy

Ultraviolet–visible (UV–vis) spectroscopy measurements of the AuNP samples were recorded on a Cary-500 spectrophotometer (Agilent Tecnologies, Santa Clara CA, USA) within the range 300 to 900 nm. The samples were prepared, using water as solvent, at 100 μg/ml. UV–vis measurements were also taken after suspension of the AuNPs in EMEM/S+ and EMEM/S- at a concentration of 100 μg/ml and at time-point 0 and 2, 4 and 24 h after incubation at 37°C.

#### Dynamic light scattering

Dynamic light scattering (DLS) was used to determine the hydrodynamic size of NPs in solution, using a Zetasizer Nano-ZS (Malvern Instruments Ltd., Worcestershire, UK). Measurements of the hydrodynamic size of the NP suspensions (100 μg/ml) in Milli-Q water and in EMEM biological medium with serum (EMEM/S+) and without serum (EMEM/S-) were taken at time 0 and at 24 h under exposure conditions (37°C and 5% CO_2_). Careful attention was paid to distinguish measurements of background serum proteins from NP agglomerates in suspensions prepared in EMEM/S+. In addition, to study stability over time and the state of particles during the cell exposure timeframe in EMEM/S-, we conducted a kinetic study. DLS measurements were taken directly after the AuNPs were suspended (time 0) and at 2, 4, 24 and 48 h of incubation in exposure conditions. Three independent analyses were performed, and the mean ± standard deviation (SD) was used to represent results. Four measurements were taken in each independent analysis, with each measurement consisting of six runs, each lasting 10 s. The average from each of these measurements was calculated using Zetasizer series software 6.20 (Malvern Instrument). The instrument was set to automatically select the best conditions for measurements. A kinetic study was not performed in EMEM/S+ because of evidence of a stable suspension from time 0 to 24 h under exposure conditions when serum was present.

#### Zeta potential

Zeta potential measurements were performed to determine the stability of the PBH-capped AuNPs in Milli-Q water and in the different medium suspensions (EMEM/S+ and EMEM/S-). A Malvern Zetasizer Nano-ZS and folded capillary cells (Malvern Instruments Ltd., Worcestershire, UK) were used. One-millilitre aliquots of AuNP suspensions (100 μg/ml) were taken directly after preparation and 24 h after incubation in the different media. Due to the limitations of high salt content in both medium suspensions, zeta potential measurements were performed only in Milli-Q water. Three independent measurements were taken, and the mean ± SD is presented.

#### Optical microscopy and visual sedimentation of AuNP suspensions

An inverted light microscope Axiovert 25 (Carl Zeiss, Madrid, Spain) equipped with a Canon EOS 1000D (Canon, Madrid, Spain) camera was used to take images. NP suspensions (0.781 to 100 μg/ml) were prepared in EMEM/S+ and EMEM/S- medium, and 100-μl aliquots of each concentration were suspended in 96-well plates. Suspensions were viewed 24 h after incubation in exposure conditions (37°C/5% CO_2_). A recent study carried out by Cho et al. [40] highlights the importance of considering sedimentation when carrying out NP toxicity studies *in vitro*. Those authors reported that different concentrations of NPs in the bottom of culture plates or ‘interaction zones’ caused by distinct ratios of sedimentation to diffusion velocities can result in variations in uptake. To detect differences in dispersion and sedimentation between the PBH-capped AuNPs in EMEM/S+ and EMEMS/S- medium, photographs were taken of the AuNP suspensions (100 μg/ml) in 1.5-ml tubes after 24-h incubation under exposure conditions.

### Cell culture and AuNP exposure

Human liver hepatocellular carcinoma cells (Hep G2) were from the American Type Culture Collection (Manassas, VA, USA). These cells were cultured in EMEM medium supplemented with 10% FBS, 1% penicillin/streptomycin, 1% ultraglutamine and 1% NEAA. They were incubated at 37°C with 5% CO_2_ in a humidified incubator. For AuNP exposure, cells were plated at densities of 7.5 × 10^4^ cells per millilitre in 96-well tissue culture microtiter plates (Greiner-Bio one, CellStar, Madrid, Spain) and subsequently incubated for 24 h. After this period, cells were exposed to a series of concentrations of the five AuNP preparations for either 2 or 24 h for ROS production studies or for 24 or 48 h for the cytotoxicity studies. AuNP suspensions were prepared in high-grade Milli-Q water to achieve a stock concentration of 1,000 μg/ml. All stock preparations were stored at 4°C. AuNP working concentrations were prepared from the 1,000 μg/ml stock preparations in EMEM/S + or EMEM/S-. Given the different size and stability profiles found for the AuNPs when suspended in these two types of medium, as shown by UV–vis, DLS and TEM analysis, we performed exposures in medium with and without serum. Working concentrations ranged from 0.781 to 100 μg/ml and were prepared using serial half dilutions. The final concentration of water in EMEM medium did not cause any osmotic imbalance. For each assay, three independent experiments were performed, with exposures carried out in triplicate for each concentration. Untreated cells in culture medium were used as negative controls in all experiments, while a serial half dilution of chloramine-T was used to produce a concentration range between 0.325 and 10 mmol/l, which was used as a positive control.

### Toxicity studies

#### Interference of AuNPs in toxicity assays

NP suspensions of each concentration tested were prepared in EMEM medium, phosphate-buffered saline (PBS) and sulfosalicyclic acid dihydrate (SSA) 5% (*w*/*v*) or MEM phenol red-free medium, depending on the assay system being used, and included in the assay as another control to check possible AuNP absorbance at the corresponding wavelengths. Very high dose-dependent interferences were observed at the wavelengths used for the methyl thiazol tetrazolium (MTT) and neutral red uptake assay (NRU) assays. Some measurements were carried out after washing the cells to determine if this washing could lead to a reduction in the number of remaining AuNPs and consequently in the interference. We also examined whether the AuNPs used in this study interacted with glutathione. For that, a cell-free experiment was set up in which a constant concentration (8 μmol/l) of glutathione was incubated with a range of AuNP concentrations for 2 h. The glutathione content was then measured as described below.

#### Cytotoxicity

##### Methyl thiazol tetrazolium and neutral red uptake assays

The MTT [(4,5-dimethylthiazoyl-2-yl)-2,5-diphenyltetrazolium bromide)] reduction assay, based on the conversion of tetrazolium salts to formazan crystals, was used to evaluate cell viability on the basis of mitochondrial activity, following the method described by Mosmann [41]. The NRU assay was used to determine the accumulation of neutral red dye in the lysosomes of viable, uninjured cells [42]. After the 24-h exposure, cells were incubated for 3 h with 500 μg/ml MTT reagent or for 2 h with 100 μg/ml neutral red dye, depending on the assay being performed. The resulting formazan crystals and remaining neutral red dye were dissolved with isopropanol or 1% glacial acetic acid in 50% ethanol, respectively. The absorbance of each well was read at 550 and 570 nm for the NRU and MTT assays, respectively, using a Tecan GENios plate reader (Tecan Group Ltd., Männedorf, Switzerland).

##### Resazurin assay

The method described by O’ Brien et al. [43], based on the reduction of resazurin to resorufin by mitochondrial oxidoreductases, was used. Cells were exposed to the AuNPs for 24 h, suspensions were removed, and cells washed with PBS and then treated with 20% (*v*/*v*) of resazurin dye reagent prepared in EMEM medium. The plate was then placed in a 37°C/5% CO_2_ incubator for 2 h, after which the fluorescence intensity was read at 532-nm excitation and 595-nm emission wavelengths using a Tecan GENios plate reader. Results are represented as a percentage of the control. To study whether there was any further reduction in viability, cytotoxicity was also analysed after 48 h of exposure.

##### Images of cell condition

At 2 and 24 h of exposure, images of the cells treated with NPs were taken and analysed for signs of cytotoxicity. An inverted light microscope (Axiovert 25, Carl Zeiss) equipped with a camera was used to take images. Evidence of cytoskeleton rounding or a change in normal shape compared to untreated controls was regarded as a sign of cytotoxicity. Also, to determine the degree of cytotoxicity, we compared the morphology of cultured cells with that of cells exposed to the positive control chloramine-T.

#### Oxidative stress

##### Quantification of reactive oxygen species

Intracellular ROS production was determined using the dichlorofluorescein (DCF) assay [44]. Stock aliquots of 2’, 7’-dichlorofluorescein diacetate (DCFH-DA) were prepared in dimethyl sulfoxide (DMSO) (100 mM) and diluted 1:1,000 in MEM phenol red-free medium to a final concentration of 100 μM, 0.1% (*v*/*v*) DMSO. After the exposure period (2 or 24 h), the medium and exposure compounds were removed, and cells were washed with PBS. Next, 100 μM of DCFH-DA probe was added to each well. The plate was incubated at 37°C/5% CO_2_ in the dark for 30 min. After the incubation period, the DCFH-DA probe was removed, and the cells were washed twice with PBS. MEM phenol red-free medium was then added to the cells, and the fluorescence was measured at 485-nm excitation and 535-nm emissions (Tecan GENios plate reader). Fluorescent readings were taken immediately (time 0) and every 15 min over 60 min, with the plates maintained under dark conditions and incubated under exposure conditions (37°C/5% CO_2_) between measurements. ROS production was calculated as the percentage increase in fluorescence per well over a 60-min period using the formula [(Ft60 − Ft0)/Ft0 × 100], where Ft60 and Ft0 are the fluorescence measured at time 60 and 0 min, respectively. This result was finally expressed as percentage of the control.

##### Reduced glutathione/oxidised glutathione ratio

The assay protocol was set up based on the optimised microtiter plate method used by Allen et al. [45]. Following the 24-h exposure, cells were lysed, and 50 μl of PBS was then added to each well. Twenty-five microlitres of cell suspension was transferred to a new 96-well plate and used to assay for protein content. To the other 25 μl of lysed cells, we added 50 μl of ice-cold 5% (*w*/*v*) SSA diluted in Milli-Q water in order to remove any interfering proteins from the sample. Of this suspension, 25 μl was used to assay for total glutathione (reduced glutathione + oxidised glutathione ratio - GSH + GSSG) content, while the other 25 μl was treated with 4-vinylpyridine 0.5 μmol/l, a scavenger of GSH, to assay the GSSG content. One hundred twenty-five microlitres of reaction buffer (PBS 143 mmol/l containing 6.3 mmol/l EDTA at pH 7.4, 229 U/ml GSH reductase, 2.39 mmol/l β-nicotinamide adenine dinucleotide phosphate (NADPH) and 0.01 mol/l 5, 5’-dithiobis (2-nitrobenzoic acid) (DTNB)) was added to each 25-μl suspension. The conversion of DTNB to 5’-thiol-2-nitrobenzoic acid (TNB) by the oxidation of GSH to GSSG was monitored by measuring absorbance at 405 nm every min over 10 min using a Tecan GENios plate reader. The rate of conversion, measured by the slope of the curve, was proportional to the concentration of glutathione in the sample. A standard curve with different concentrations of GSSG was used to calculate the glutathione contents in the samples.

#### Statistical analysis

For all the assays used, we performed three independent experiments with exposures carried out in triplicate for each concentration. The values shown are expressed as mean ± standard error of the mean (SEM). Sigma Plot 12 software (Systat Software Inc, CA, USA) was used for statistical analysis. The normality of the distribution was checked by means of the Shapiro-Wilk test. Equal variance was not assumed by the software and was tested (*F* test). A one-way repeated measures analysis of variance (RM-ANOVA) was carried out, followed by a *post hoc* Dunnett’s test with *P* < 0.05 or *P* < 0.01.

## Results

### Physico-chemical characterisation of PBH-capped AuNPs

**Figure 2:**
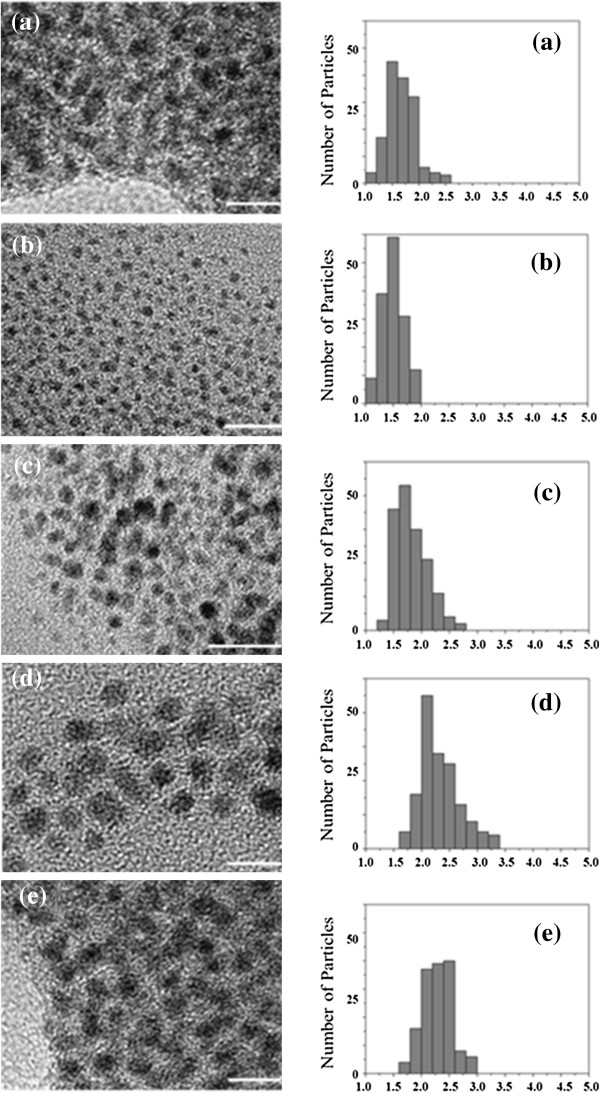
**TEM images and size histograms of PBH-capped AuNPs. (a)** Au[(Gly-Trp-Met)_2_B], **(b)** Au[(Gly-Tyr-TrCys)_2_B], **(c)** Au[(Gly-Tyr-Met)_2_B], **(d)** Au[(Met)_2_B] and **(e)** Au[(TrCys)_2_B] [Scale bars: 10 nm for **(a)** and **(b)**; and 5 nm for **(c)**, **(d)** and **(e)**].

The AuNPs were synthesised using PBHs as capping ligands (Figure 1). In a previous study [9], we used PBHs containing cysteine (Cys), tyrosine (Tyr) and glycine (Gly) amino acids to form stable AuNPs: Au[(TrCys)_2_B] and [(Gly-Tyr-TrCys)_2_B]. In the present study, we demonstrate that the amino acids methionine (Met) and tryptophan (Trp) are also useful to prepare stable functionalised AuNPs such as Au[(Met)_2_B], Au[(Gly-Tyr-Met)_2_B] and Au[(Gly-Trp-Met)_2_B]. TEM images of the PBH-capped AuNPs and the corresponding size distribution histograms are shown in Figure 2. The micrographs show isolated near-spherical NPs with diameters of 1.5, 1.6, 2.3, 1.8 and 2.3 nm for Au[(Gly-Tyr-Met)_2_B], Au[(Gly-Trp-Met)_2_B], Au[(Met)_2_B], Au[(Gly-Tyr-TrCys)_2_B] and Au[(TrCys)_2_B], respectively. The NPs stabilised with the bulkiest PBHs were smaller. This observation may be attributable to the steric bulk of the ligand controlling NP growth.

**Figure 3:**
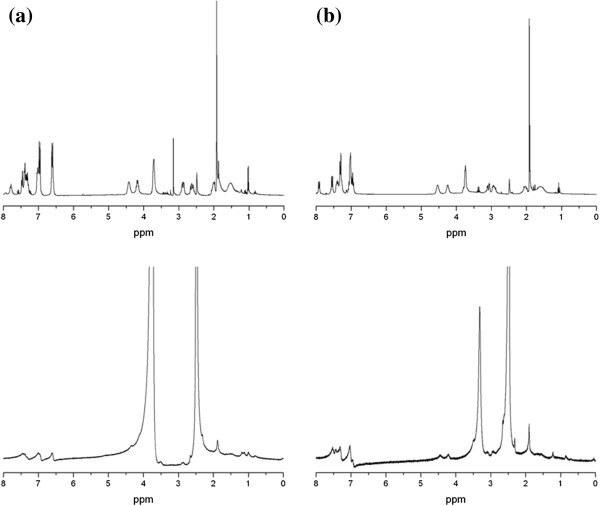
^**1**^**H NMR spectra of free PBHs and PBH-capped AuNPs. (a)** Free PBH (Gly-Tyr-Met)_2_B (top) and ^1^H NMR spectrum of AuNP Au[(Gly-Tyr-Met)_2_B] (bottom) in DMSO-*d*6, and **(b) **^1^H NMR spectrum of free PBH (Gly-Trp-Met)_2_B (top) and ^1^H NMR spectrum of AuNP Au[(Gly-Trp-Met)_2_B] (bottom) in DMSO-*d*6.

TEM images combined with elemental analysis were used to estimate the molecular formula of the PBH-capped AuNPs (Table 1). The AuNPs prepared with PBHs containing Met residue were stabilised with a lower number of ligands on each AuNP surface compared to the AuNPs capped with other PBH ligands. A direct comparison of Au[(Met)_2_B] and Au[(TrCys)_2_B] revealed fewer ligands for the Met-containing PBH-AuNP, despite both having the same diameter. ^1^H NMR spectra and FT-IR absorption spectra of free PBHs and of the PBH-capped AuNPs were measured to identify the interactions between the gold surface and the capping ligand. The NMR spectra of the AuNPs showed broad signals compared to the free PBHs. Figure 3 shows ^1^H NMR spectra of Au[(Gly-Tyr-Met)_2_B] and its free PBH (Gly-Tyr-Met)_2_B in DMSO-*d*_6_. The signal of the H-α of the Met residue appeared at approximately 1.5 ppm in the PBH (Gly-Tyr-Met)_2_B NMR spectrum and was significantly broadened in that of Au[(Gly-Tyr-Met)_2_B]. A similar line broadening was also observed in the NMR spectrum of Au[(Gly-Trp-Met)_2_B] (Figure 3) and of Au[(Met)_2_B] (see Additional file 2: Figure S1). These observations indicate that the PBH was attached to the gold surface through the Met residue [46]. Analogous results were observed for the NMR spectra of Au[(Gly-Tyr-TrCys)_2_B] and Au[(TrCys)_2_B] [9], where the sulphur atom of the TrCys residue is involved in the surface binding.

**Table 1:** Structural characteristics of the AuNPs from elemental analysis and TEM data

**AuNP**	**Size (nm)**	**Calculated m/n**^**a **^**from %N**^**b**^	**Number of Au atoms**^**c**^	**PBH units per Au nanoparticle**	**Mw**
Au[(Gly-Trp-Met)_2_B]	1.6	0.062	126	8	32,106
**Au[(Gly-Tyr-TrCys)**_**2**_**B]**	1.8	0.22	180	40	90,397
Au[(Gly-Tyr-Met)2B]	1.5	0.064	104	7	27,100
Au[(Met)_2_B]	2.3	0.154	375	57	102,625
Au[(TrCys)_2_B]	2.3	0.26	375	97	164,377

Bold emphasis is used to signal the most stable AuNP; ^a^*m*, Number of PBH units; *n*, Number of Au atoms; ^b^%N from elemental analysis; ^c^estimated supposing spherical particles and applying *N* = 30.89602 D3 [47].

**Figure 4:**
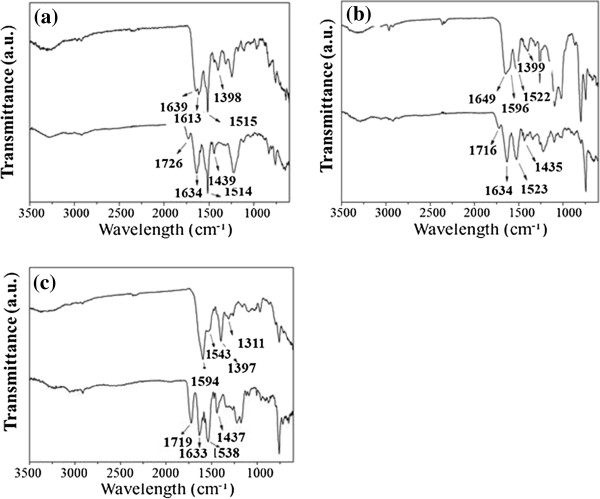
**FT-IR spectra for free PBHs and PBH-capped AuNPs. (a)** Free PBH (Gly-Tyr-Met)_2_B (bottom) and AuNP Au[(Gly-Tyr-Met)_2_B] (top), **(b)** free PBH (Gly-Trp-Met)_2_B (bottom) and AuNP Au[(Gly-Trp-Met)_2_B] (top) and **(c)** free PBH (Met)_2_B (bottom) and AuNP Au[(Met)_2_B] (top).

The FT-IR spectra are shown in Figure 4. For Au[(Gly-Tyr-Met)_2_B], Au[(Gly-Trp-Met)_2_B] and Au[(Met)_2_B], the band caused by the C = O stretching mode of the carboxylic group was absent. However, two bands were observed around 1,600 and 1,398 cm^−1^, assigned to the asymmetric and symmetric stretching vibrations of carboxylate anions [48]. These results suggest that the carboxylic groups are also involved in PBH interactions with the gold surface. Significant changes were observed in the amide I band in the spectra of capped NPs compared with those of the free PBHs. For Au[(Gly-Tyr-Met)_2_B] and Au[(Gly-Trp-Met)_2_B], the amide I band red shifted to 1,639 and 1,649 cm^−1^, respectively, however, the amide band II appeared in the same position at 1,515 and 1,523 cm^−1^, respectively. For Au[(Met)_2_B], the band assigned to amide I blue shifted to about 1,600 cm^−1^ and the amide II band red shifted to 1,543 cm^−1^. These findings indicate that conformational changes occur in the structure of the capping ligands attached to the NPs. Similar conclusions were drawn from the IR spectra of Au[(Gly-Tyr-TrCys)_2_B] and Au[(TrCys)_2_B] [9].

### Physico-chemical characterisation of PBH-capped AuNPs under culture conditions

#### UV–vis absorption spectroscopy

**Figure 5:**
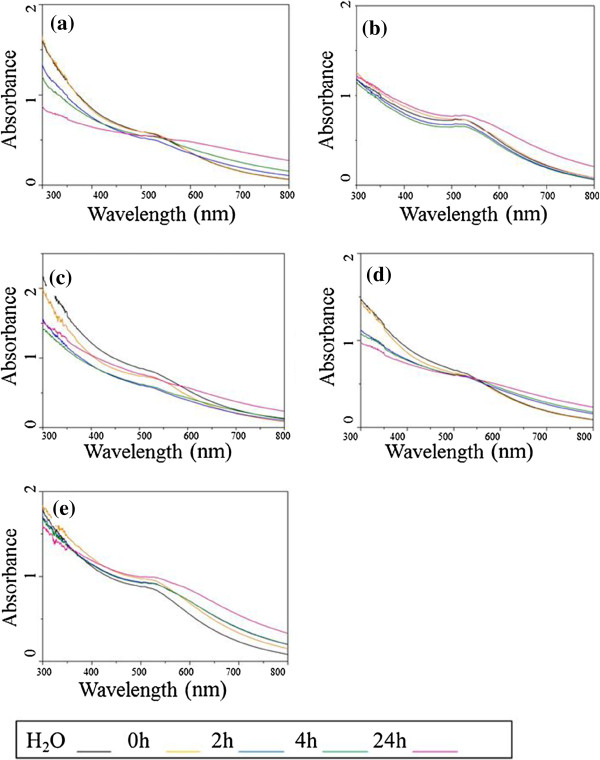
**UV–vis absorption spectra of AuNPs. (a)** Au[(Gly-Tyr-Met)_2_B], **(b)** Au[(Gly-Tyr-TrCys)_2_B], **(c)** Au[(Gly-Trp-Met)_2_B], **(d)** Au[(Met)_2_B] and **(e)** Au[(TrCys)_2_B], in water and EMEM/-, each at a concentration of 100 μg/ml and at time point 0 and 2, 4 and 24 h of incubation at 37°C.

Figure 5 shows the UV–vis absorption spectra of AuNPs in Milli-Q water at time 0 and in EMEM/S- taken at different time points under assay conditions (37°C and 5% CO_2_). The spectrum in water, at a concentration of 100 μg/ml, shows the surface plasmon resonance (SPR) band in the range of 505 to 519 nm, characteristic of colloidal gold. The position of the SPR band was established as a function of particle size, stabilising ligand and solvent dielectric [49]. The SPR band of the UV–vis spectra of AuNPs (100 μg/ml) in EMEM/S- changed over time. The UV–vis spectra of the AuNPs after 24-h incubation showed a slight broadening of the SPR band, in the range of 550 to 800 nm, indicating the aggregation of NPs in EMEM/S- medium as a result of the presence of salts in the medium. The band was also red shifted to 525 nm, in the case of Au[(Gly-Trp-Met)_2_B] and Au[(Gly-Tyr-Met)_2_B], and close to 560 nm for Au[(Gly-Tyr-TrCys)_2_B], Au[(Met)_2_B] and Au[(TrCys)_2_B]. The red shift of the SPR band can be induced by a change in the refractive index that surrounds the AuNPs or by aggregation of NPs [50] caused by the presence of chemical or biological analytes in the culture medium. In addition, in the case of Au[(Gly-Trp-Met)_2_B], Au[(Gly-Tyr-Met)_2_B] and Au[(Met)_2_B], which contain methionine, a minimal decrease in the intensity band was observed over time. This decrease was associated with the structure and optical properties of gold. The amino acids of the culture medium were adsorbed on the surface of Au[(Gly-Trp-Met)_2_B], Au[(Gly-Tyr-Met)_2_B] and Au[(Met)_2_B], and this effect might mask the optical absorption of these NPs [51]. AuNPs containing methionine were stabilised with a lower number of ligands and may have the capacity to link more molecules of amino acid on their surfaces. In comparison, the UV–vis spectra of AuNPs in EMEM/S+ (100 μg/ml) (see Additional file 3: Figure S2) did not show any change in the range of 550 to 800 nm. These spectra revealed no noticeable aggregation in preparations of AuNPs in EMEM/S+. Nevertheless, some decreases in the band intensities occurred over time in all cases, thereby indicating the adsorption of serum proteins from the medium [52].

#### Zeta potential

To study changes in AuNP stability, on the basis of electrostatic interaction, zeta potential measurements were performed. Due to the high salt content of EMEM/S+ and EMEM/S- media, measurements were performed only in Milli-Q water. Measurements were taken just after preparation of AuNP suspensions (100 μg/ml), at initial time (T0) and 24 h after incubation under assay conditions. The five AuNP preparations used in this study, namely Au[(Gly-Trp-Met)_2_B], Au[(Gly-Tyr-TrCys)_2_B], Au[(Gly-Tyr-Met)_2_B], Au[(Met)_2_B] and Au[(TrCys)_2_B], showed zeta potentials of −31.6 ± 2.02, −37 ± 1.04, −36 ± 1.12, −39 ± 1.07 and −43.3 ± 1.13 mV, respectively (Table 2). All zeta potentials were negative and remained negative over time.

**Table 2:** Physico-chemical properties of PBH-capped AuNPs (100 μg/ml) under different conditions over time

	**Milli-Q water**			**EMEM/S+**		**EMEM/S-**	
	**T0**	**T24**	**T0**	**T0**	**T24**	**T0**	**T24**
**AuNP**	**Size**^**a**^	**Size**	**Zeta**^**b**^	**Size**	**Size**	**Size**	**Size**
	nm	nm	mV	nm	nm	nm	nm
Au[(Gly-Trp-Met)_2_B]	148 ± 2	148 ± 1	−31.6 ± 2.0	242 ± 4	243 ± 6	233 ± 15	1,239 ± 26
**Au[(Gly-Tyr-TrCys)**_**2**_**B]**	143 ± 1	143 ± 1	−37 ± 1.4	261 ± 1	261 ± 2	251 ± 15	195 ± 2
Au[(Gly-Tyr-Met)_2_B]	591 ± 73	507 ± 65	−36 ± 1.1	987 ± 205	987 ± 207	407 ± 21	1,230 ± 8
	161 ± 5	150 ± 12		203 ± 13	201 ± 9		
Au[(Met)_2_B]	229 ± 23	228 ± 10	−39 ± 1.1	190 ± 13	190 ± 4	1568 ± 28	1,368 ± 25
	38 ± 6	40 ± 3		27 ± 9	28 ± 3		
Au[(TrCys)_2_B]	205 ± 1	205 ± 1	−43.2 ± 1.1	261 ± 3	260 ± 4	271 ± 23	908 ± 23
							97 ± 3

T0 represents measurements directly after preparation and T24 measurements 24 h after incubation under cell exposure conditions (37°C, 5% CO_2_). Average values of three independent measurements are presented (mean ± SD). Bold emphasis is used to signal the most stable AuNP; DLS, dynamic light scattering. ^a^Hydrodynamic size (Size); ^b^zeta potential (Zeta) of AuNPs in Milli-Q water.

**Figure 6:**
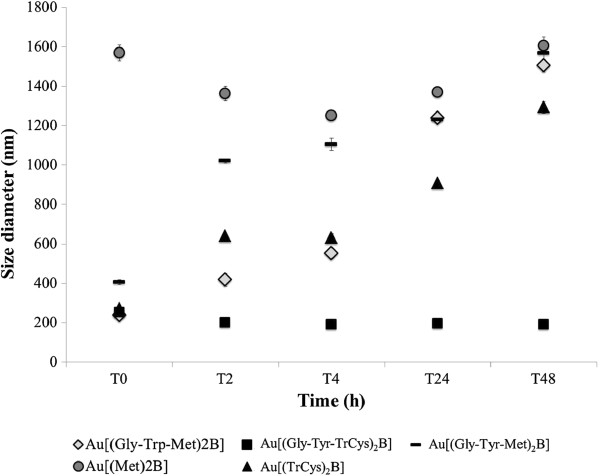
**Size distribution of the PBH-capped AuNP preparations (100 μg/ml) in EMEM/S- over time using DLS.** Maximum intensity hydrodynamic diameter (nm) measured directly after preparation (T0) and at 2 h (T2), 4 h (T4), 24 h (T24) and 48 h (T48) of incubation are shown.

DLS was used to measure the hydrodynamic diameters of NPs in Milli-Q water and in medium suspension (100 μg/ml). DLS measurements were taken just after suspension (T0) and after 24 h incubations (T24) under assay conditions. In water, all AuNP preparations formed agglomerates, showing characteristic maximum intensity hydrodynamic diameters of ≤200 nm (Table 2). The Au[(Gly-Tyr-Met)_2_B] also appeared as larger agglomerates, with a maximum intensity diameter of 591 nm at time 0, while Au[(Met)_2_B] presented an additional NP population of only 38 nm in diameter. Using the size distribution of the AuNPs in water as a reference, we observed an increase in hydrodynamic size for all the AuNP preparations when incubated in EMEM/S+ and EMEM/S-, but to different extents. The average increase in hydrodynamic size for all the NP preparations in EMEM/S+ was 85 ± 26 nm at time 0 (Table 2). An exception was AuNPs Au[(Met)_2_B], which did not increase in size in this medium. Also, with respect to the other three NPs, the larger agglomerates of Au[(Gly-Tyr-Met)_2_B] underwent a much larger increase in size from 591 to 987 nm. The hydrodynamic sizes of Au[(Gly-Trp-Met)_2_B] in water and EMEM/S+ are noticeably smaller than found for Au[(Gly-Tyr-Met)_2_B]. These differences could be attributed to the presence, in the PBH ligand (Gly-Trp-Met)_2_B, of the additional anchoring site, indole NH group of the Trp reside, which may be contributing to the stabilisation of this nanoparticle. All AuNP preparations remained in the same state in water and EMEM/S+ over 24 h, with no change in their size distribution profiles from those measured directly after preparation (Table 2). In contrast, for AuNPs incubated in EMEM/S-, a time-dependent increase in size was detected (Table 2). At time 0 (T0), the average increase in size in EMEM/S- was 86 ± 21 nm, similar to the distribution of most PBH-capped NPs in EMEM/S+, except Au[(Met)_2_B], which experienced extensive agglomeration at time 0 (1,568 nm) with smaller fluctuations in its maximum hydrodynamic diameter over 24 h in EMEM/S- (1,368 nm). The Au[(Gly-Trp-Met)_2_B], Au[(Gly-Tyr-Met)_2_B] and Au[(TrCys)_2_B] showed a time-dependent increase in size distribution, represented by agglomerates of 1,239, 1,230 and 908 nm after 24 h of incubation, respectively (Table 2). Au[(Gly-Tyr-TrCys)_2_B] was the only preparation of AuNP that remained in the same relative size distribution profile over time and with the same maximum intensity hydrodynamic diameter (±54 nm) after a 24-h incubation in EMEM/S-. A kinetic study was performed to monitor changes in the AuNP suspensions (100 μg/ml) over time (Figure 6). DLS measurements were taken just after NP suspension in EMEM/S- and after 2-, 4-, 24- and 48-h incubations under assay conditions. The size distribution profiles for each preparation in EMEM/S- at each time point are represented in Figure 6, which shows an increasing tendency of agglomeration for all the AuNPs, except Au[(Gly-Tyr-TrCys)_2_B], which remained stable over time.

### Transmission electron microscopy

**Figure 7:**
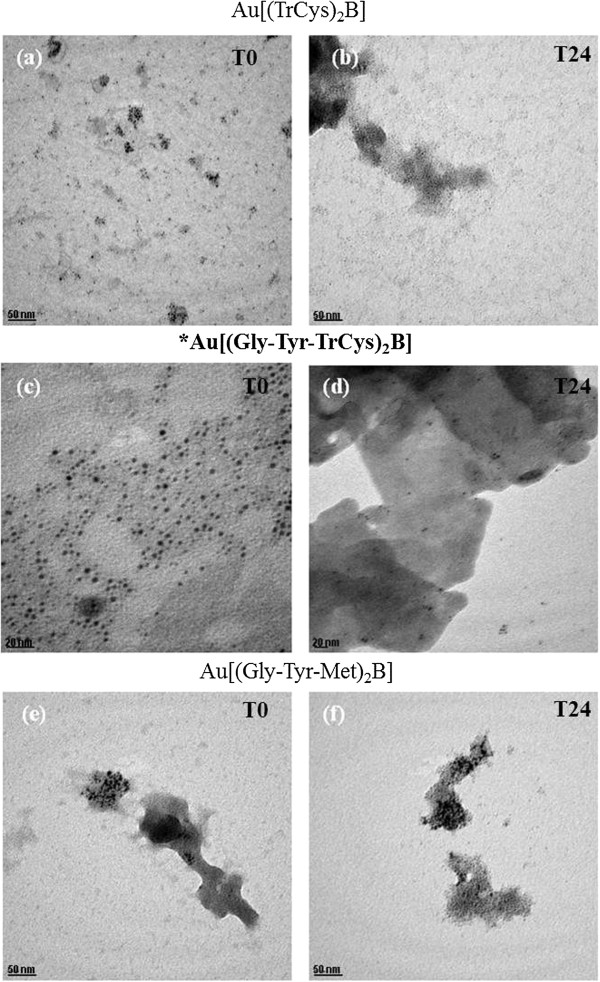
**TEM images of AuNPs in EMEM/S- after preparation. (a)** Au[(TrCys)_2_B], **(c)** Au[(Gly-Tyr-TrCys)_2_B] and **(e)** Au[(Gly-Tyr-Met)_2_B], and at 24 h of incubation; **(b)** Au[(TrCys)_2_B], **(d)** Au[(Gly-Tyr-TrCys)_2_B] and **(f)** Au[(Gly-Tyr-Met)_2_B] [Scale bar **(c)** and **(d)** is 20 nm, and for all other images, scale bar is 50 nm]; asterisk and bold letters are used to signal the most stable AuNP.

Transmission electron micrographs were taken of the PBH-capped AuNPs after suspension in EMEM/S- medium (T0) and after 24 h of incubation (T24) under assay conditions (37°C/5% CO_2_). Representative TEM images of Au[(Gly-Tyr-TrCys)_2_B], Au[(TrCys)_2_B] and Au[(Gly-Tyr-Met)_2_B] are shown in Figure 7. Figure 7a,c shows TEM micrographs of Au[(TrCys)_2_B] and Au[(Gly-Tyr-TrCys)_2_B] directly after suspension, respectively. Both images reveal isolated NPs with the same size (1 to 3 nm) in the absence of medium. After 24 h of incubation, Au[(Gly-Tyr-TrCys)_2_B] and Au[(TrCys)_2_B] (Figure 7b,d) showed agglomeration and a clear interaction of individual NPs with medium components, as determined from TEM images. By comparing the micrographs, the highest degree of agglomeration in the case of Au[(Gly-Tyr-Met)_2_B] (Figure 7e,f) after suspension in medium can be appreciated. Therefore, one would expect the surface chemistry of these NPs upon interaction with media not to be the same as for the NPs initially prepared [53].

### Optical microscopy and visual sedimentation of AuNP suspensions

**Figure 8:**
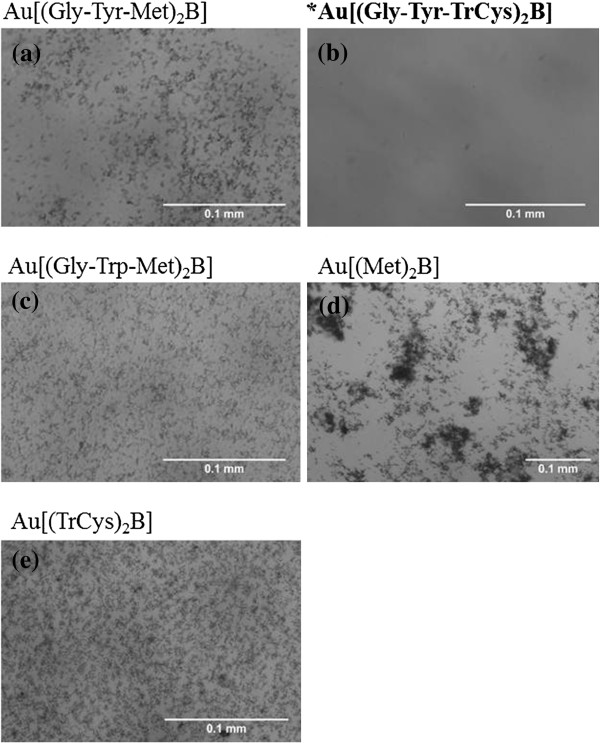
**PBH-capped AuNPs (100 μg/ml) after 24-h incubation in EMEM/S- as viewed using optical microscope. (a)** Au[(Gly-Trp-Met)_2_B], **(b)** Au[(Gly-Tyr-TrCys)_2_B], **(c)** Au[(Gly-Tyr-Met)_2_B, **(d)** Au[(Met)_2_B and **(e)** Au[(TrCys)_2_B]; asterisk and bold letters are used to signal the most stable AuNP.

Large distinctive agglomerates of micrometre scale were observed for all AuNP preparations when viewed under an optical microscope (Figure 8), with the exception of Au[(Gly-Tyr-TrCys)_2_B] (Figure 8b). Also upon visual observation of the AuNP suspensions in the different medium suspensions after 24 h of incubation, we made some key observations regarding sedimentation over time. After 24 h of incubation in EMEM/S-, Au[(Gly-Trp-Met)_2_B], Au[(Gly-Tyr-Met)_2_B], Au[(Met)_2_B] and Au[(TrCys)_2_B] sedimented out of solution, as determined by the presence of a pellet at the bottom of the tubes. Au[(Gly-Tyr-TrCys)_2_B] remained dispersed in solution, having a visibly darker appearance in suspension. In the case of the serum-containing medium, EMEM/S+, sedimentation was less apparent. AuNP Au[(Gly-Tyr-TrCys)_2_B], along with Au[(Met)_2_B] and Au[(TrCys)_2_B], had a visibly darker appearance, thereby suggesting different dispersion rates for these particles when serum was present.

### Toxicity studies

#### Interference of AuNPs with toxicity assays

**Figure 9:**
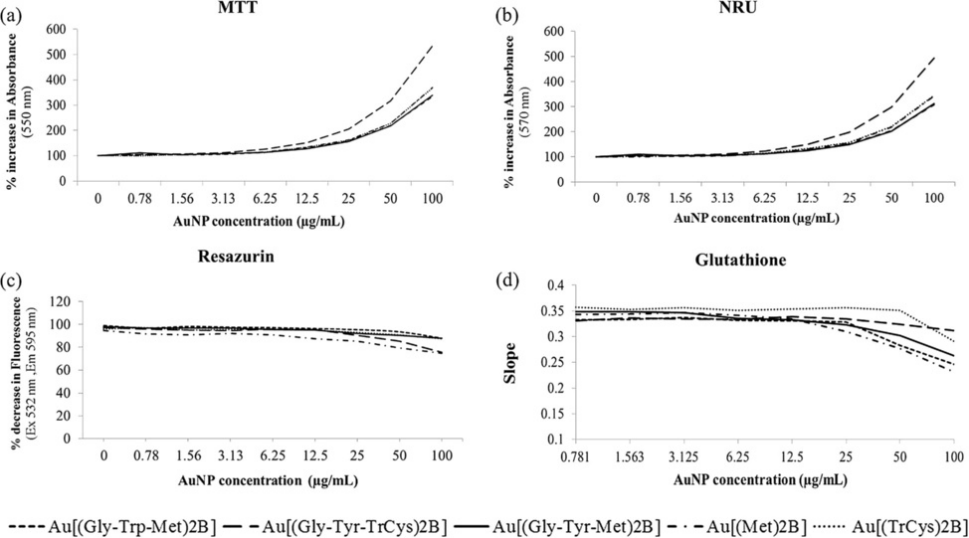
**PBH-capped AuNP interference with the toxicity assays. (a)** MTT, **(b)** neutral red uptake (NRU), **(c)** resazurin-based cytotoxicity assay and **(d)** glutathione detection.

AuNP concentration-dependent interference was detected with the toxicity assays used in this study (Figure 9). In the case of the commonly used MTT and NRU assays, absorbance is used as the assay readout. Concentration-dependent interference by control samples containing AuNPs without cells was observed at both of the wavelengths used, 570 and 550 nm, as a result of the absorbance of AuNPs at the same wavelengths (Figure 9a,b). A concentration-dependent increase in absorbance levels was evident from a 6.25 μg/ml exposure concentration, which reached a 500% increase at the highest concentration used in this study (100 μg/ml) for both wavelengths. The removal of the media and washing with PBS did not lead to a significant reduction in interferences to levels that permitted the assays to be used appropriately. To combat interferences seen using absorbance as endpoint readout, a cytotoxicity assay using resazurin and its fluorescent product was applied. AuNP-only controls suspended in EMEM medium were included and interference was detected. We observed a concentration-dependent decrease in the levels of fluorescence as a result of AuNP interference (Figure 9c). At the highest concentration of AuNP, levels decreased by 11% to 24% depending on the AuNP in question. Au[(Gly-Tyr-TrCys)_2_B] exhibited the highest level of interference. The results were interpreted with care in order to avoid drawing erroneous conclusions. Cytotoxicity was assumed only when the decrease in fluorescence was lower than possible interference levels. We also examined whether the AuNPs used in this study interacted with the glutathione assay. AuNPs absorbed at the wavelength used in this assay (405 nm). A dose-dependent increase appeared for some of them at concentrations of 1.56 μg/ml (data not shown) or higher. Additionally, when glutathione was incubated with a range of AuNP concentrations for 2 h the level of free glutathione decreased as the concentration of AuNPs increased (Figure 9d). Therefore, this assay was not considered suitable for studying the oxidative stress potential of the AuNPs. However, no interference was observed with the ROS production assay (data not shown).

#### Cytotoxicity

##### Methyl thiazol tetrazolium and neutral red uptake assays

The MTT and NRU assays could not be performed as there was AuNP interference at the wavelengths used in these tests (570 and 550 nm, respectively) (Figure 9a,b).

##### Resazurin assay

Cytotoxicity assays were performed with cells incubated in EMEM/S+ and EMEM/S- after 24- and 48-h exposure periods. Only results with cells incubated in EMEM/S- are shown in Table 3, as clear evidence of cytotoxicity in cells exposed to AuNPs in EMEM/S+ could not be determined because of high interference levels in this assay under these conditions (Figure 9c). Cytotoxicity is expressed as percentage of live cells (viability) compared to the untreated control (100%). At the highest concentration (100 μg/ml), all AuNP preparations caused approximately 10% decrease in viability. This was the highest decrease in viability recorded after 24 h of incubation for the AuNP preparations tested. This decrease in viability was not higher than that recorded for the cell-free AuNP-only controls in the interference studies (11% to 24% decrease). Therefore, the reduction in viability is perceived to be a result of NP interference and cannot be reported as cytotoxicity. After 48 h of incubation, the level of cytotoxicity for Au[(Gly-Tyr-Met)_2_B], Au[(Met)_2_B] and Au[(TrCys)_2_B] increased significantly for the two highest doses of 50 and 100 μg/ml (*p* < 0.01). Viability was reduced to levels beyond which AuNP interference may be responsible. A significant decrease (*p* < 0.01) in cell viability was observed for the AuNP Au[(Gly-Trp-Met)_2_B] only at the highest dose (100 μg/ml). Exposure to AuNP Au[(Gly-Tyr-TrCys)_2_B] also resulted in a reduction in viability over time but not below interference levels. This observation thus suggests that this AuNP presents increased biocompatibility.

**Table 3:** Cytotoxicity of PBH-capped AuNPs following 24- and 48-h exposure (EMEM/S-), using resazurin assay

		**Exposure concentration (μg/ml)**			
**Exposure duration**	**AuNP**	**12.5**	**25**	**50**	**100**
Au[(Gly-Trp-Met)_2_B]	24 h	97 ± 1	97 ± 1*	96 ± 1*	94 ± 0.3**^a^
Viability (%)	48 h	98 ± 1	98 ± 2	91 ± 1	69 ± 4**^a^
Measured interference (%)		96 ± 2	95 ± 2	94 ± 4	88 ± 4
**Au[(Gly-Tyr-TrCys)**_**2**_**B]**	24 h	98 ± 1	96 ± 1*	93 ± 1**	90 ± 1**
Viability (%)	48 h	95 ± 2	100 ± 2	95 ± 3	87 ± 2*
Measured interference (%)		96 ± 3	90 ± 6	85 ± 7	76 ± 6
Au[(Gly-Tyr-Met)_2_B]	24 h	96 ± 1	96 ± 1*	96 ± 1*	91 ± 2**^a^
Viability (%)	48 h	94 ± 1	91 ± 6*	81 ± 6**	71 ± 5**^a^
Measured interference (%)		95 ± 2	92 ± 2	90 ± 4	88 ± 4
Au[(Met)_2_B]	24 h	97 ± 1	96 ± 0.4*	93 ± 0.4**	94 **±** 2**^a^
Viability (%)	48 h	97 ± 1	91* ± 3	88 ± 4**	68 ± 4 **^a^
Measured interference (%)		93 ± 1	91±	91 ± 2	89 ± 5
Au[(TrCys)_2_B]	24 h	98 ± 1	97 ± 1	92 ±2*	88 ± 1**
Viability (%)	48 h	94 ± 4	93 ± 1	88 ± 2 **	77 ± 1**
Measured interference (%)		95 ± 1	93±	91 ± 3	87 ± 4

Also shown are the measured interferences in percent (%) of the control. Average values of three independent measurements are presented (mean ± SEM). Bold emphasis is used to signal the most stable AuNP; *P < 0.05 and **P < 0.01, significant differences from control values. ^a^Significant differences between response to 24- and 48-h exposure.

##### Images of cell condition

**Figure 10:**
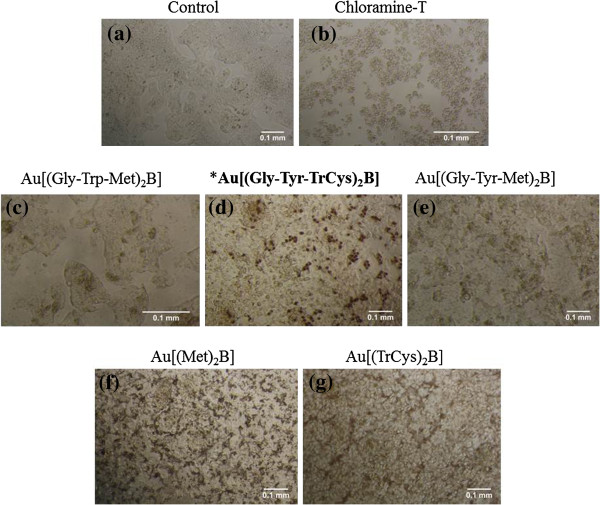
**Optical microscope images of the morphology of Hep G2 cells. (a)** untreated **(b)** after 24-h incubation with chloramine-T (positive control) and after 24-h exposure to AuNP preparations **(c)** Au[(Gly-Trp-Met)_2_B], **(d)** Au[(Gly-Tyr-TrCys)_2_B], **(e)** Au[(Gly-Tyr-Met)_2_B], **(f)** Au[(Met)_2_B] and **(g)** Au[(TrCys)_2_B] in EMEM/S-; asterisk and bold letters are used to signal the most stable AuNP.

An optical microscope was used to view the cells and NPs in EMEM/S- at various time points throughout the exposure. The study was performed only for exposures using EMEM/S- because of evidence of higher instability and toxicity of AuNPs under these conditions. Figure 10 shows Hep G2 cells after 24 h of incubation with NP concentrations of 100 μg/ml. The AuNPs Au[(Met)_2_B] formed large agglomerates that covered almost the entire well (Figure 10f). While this phenomenon made it difficult to view the cells, evidence of cell rounding was observed when compared to the untreated cells (Figure 10a). However, the cells most dramatically affected were those exposed to Au[(Gly-Tyr-TrCys)_2_B] and Au[(TrCys)_2_B] (Figure 10d,g, respectively). Unique and distinct dark assemblages in the cells exposed to Au[(Gly-Tyr-TrCys)_2_B] (Figure 10d) were evident. The size of Au[(Gly-Tyr-TrCys)_2_B] agglomerates did not permit NP visualisation in a cell-free Au[(Gly-Tyr-TrCys)_2_B] suspension (Figure 8). This observation led us to believe that the assemblies, visible when Au[(Gly-Tyr-TrCys)_2_B] was in contact with cells (Figure 10d), are a result of cell damage or are formed from cellular interaction with these AuNPs. The cells exposed to Au[(TrCys)_2_B] (Figure 10g) showed the characteristic appearance of cells treated with chloramine-T (Figure 10b), the chemical used in this study as a positive control. The cells rounded completely into a blister-like structure. However, the AuNPs did not appear to interact with the cells and instead were suspended in the medium. The morphology of Hep G2 cells incubated with Au[(Gly-Trp-Met)_2_B] was comparable with that of untreated cells, despite the presence of some dark assemblages (Figure 10c). Cells exposed to Au[(Gly-Tyr-Met)_2_B] (Figure 10e) also seemed to retain healthy cellular features, with NPs settled on clear areas of the 96-well plate, thereby suggesting limited NP-cellular interaction.

#### Oxidative stress

##### Quantification of reactive oxygen species

**Figure 11:**
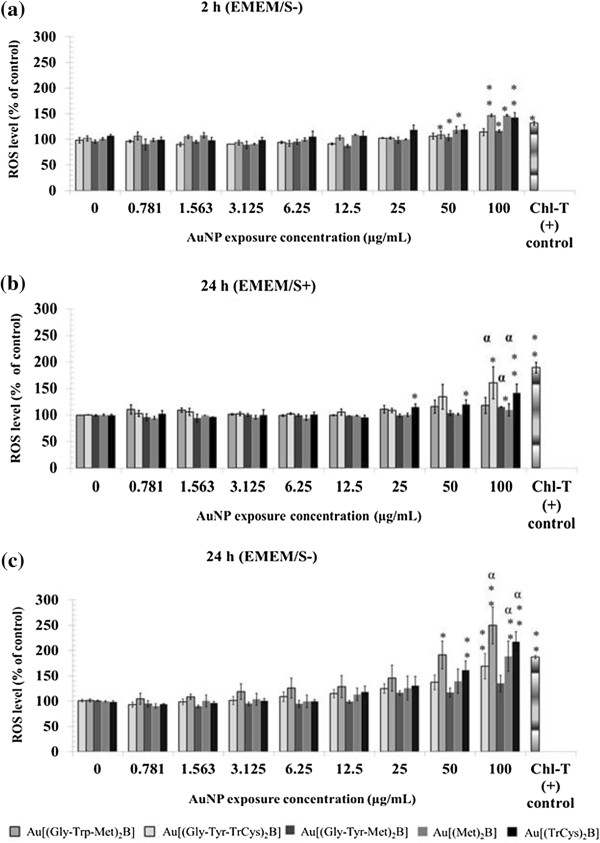
**Comparison of oxidative stress response in Hep G2 cell line. (a)** Two and **(b)** 24 h of exposure to AuNP under EMEM/S- and **(c)** after 24 h of exposure to EMEM/S+ assay conditions. Average values of three independent measurements are presented (mean ± SEM). Significant differences from control values are shown (*P < 0.05, **P < 0.01). α indicates significant differences between responses, as shown by pair-wise comparison analysis.

A concentration-dependent increase in ROS in Hep G2 cells exposed to the two highest doses (50 and 100 μg/ml) of AuNPs in EMEM/S- was evident and significant as early as 2 h and increased after 24 h of exposure (Figure 11a,b). Exposure to Au[(Gly-Tyr-TrCys)_2_B] for 24 h produced the highest increase in ROS levels, showing a 150% increase after exposure to the highest concentration tested (100 μg/ml) (Figure 11b). Au[(Gly-Tyr-Met)_2_B] showed the lowest oxidative potential, with only a 40% increase in ROS level after 24 h of exposure. Exposure assays after 24 h using EMEM/S+ (Figure 11c) led to a reduction in ROS production in Hep G2 cells in comparison with EMEM/S- for all AuNP preparations after the same period. Most dramatically, the capacity of Au[(Gly-Trp-Met)_2_B] and Au[(Met)_2_B] to elicit ROS generation disappeared while the ability of Au[(Gly-Tyr-TrCys)_2_B], Au[(Gly-Tyr-Met)_2_B] and Au[(TrCys)_2_B] to elicit an oxidative stress response was attenuated, with a significant difference in responses, as measured statistically.

##### Reduced glutathione/oxidised glutathione ratio

This assay could not be performed due to AuNP interference with the system (Figure 9d). There is a concentration-dependent decrease in the rate of conversion (slope) of DTNB to TNB caused by the interaction of the AuNPs with glutathione.

## Discussion

In this study, we have made some important observations concerning the biological behaviour of PBH-capped AuNPs. Depending on the structure of the PBH capping ligand, the behaviour of AuNPs differed both in terms of stability and biocompatibility. The PBH-capped AuNPs used in this study associated in different ways, forming agglomerates of different sizes under culture conditions, as demonstrated through DLS measurements, UV–vis analysis and optical imaging. The stability of these particles over time is dictated by both the structure of the PBH ligand and the surrounding medium. Even the smallest of changes in ligand structure can lead to great differences in AuNP behaviour. We detected clear differences in the hydrodynamic size of AuNPs in EMEM/S+ and EMEM/S-. In the former, all the AuNP preparations experienced a uniform increase in hydrodynamic size, possibly because of serum coating forming a corona, as proposed for other NPs [54,55], but these preparations remained in a stable size distribution over 24 h. It would appear that the serum coating prevented further interaction between the individual AuNPs over time. In agreement with this finding, Ehrenberg et al. [56] demonstrated protein binding to polystyrene particles (100 nm) with COOH functional groups within seconds with stable protein-coated NPs after as little as 30 min and these NPs remained stable for the entire test period (4 h). According to our UV–vis and DLS analyses, all PBH-capped AuNPs form stable agglomerates under culture conditions when serum was present. However, considerations are needed when serum is not present. In this case, the structure of the PBH greatly influences the stability and biocompatibility of the AuNP. In EMEM/S-, the characteristic hydrodynamic size distribution profiles of all the NP preparations increased considerably in a time-dependent manner, with the exception of Au[(Gly-Tyr-TrCys)_2_B]. This PBH-capped AuNP had the same hydrodynamic size distribution profile range (150 to 260 nm) in EMEM/S- as in a water suspension and in medium containing serum. Thus, the hydrodynamic size decreased approximately 40 nm upon incubation. This reveals that the medium culture had less of an effect on the AuNPs Au[(Gly-Tyr-TrCys)_2_B]. Interestingly, sizes up to micron scale were recorded for Au[(Met)_2_B] (1,568 nm) almost immediately upon contact with the EMEM/S- medium. UV–vis analysis of this AuNP suspension over time revealed red shifts in the SPR band, with a slight broadening, suggesting agglomeration of NPs in that medium. For Au[(Gly-Trp-Met)_2_B], Au[(Gly-Tyr-Met)_2_B] and Au[(Met)_2_B], which contain methionine, a minimal decrease in the intensity band was observed over time, probably caused by the adsorption of amino acids of the culture medium. In contrast, in the UV–vis spectrum of Au[(Gly-Tyr-TrCys)_2_B], the decrease in the intensity of SPR band was not observed, suggesting that the steric bulk and the strong interaction of (Gly-Tyr-TrCys)_2_B with the gold prevents the adsorption of compounds from culture medium. Only after 24-h incubation, the UV–vis spectrum shows a shoulder in the range of 550 to 800 nm. It seems that the aggregation process occurs slower than in other samples. AuNP agglomeration and interaction with medium over time was also confirmed with TEM analysis.

Differences in the structure of the PBH capping agents used in this study led to distinct associations between individual AuNPs and their environment. The stability of Au[(Gly-Tyr-TrCys)_2_B] and Au[(Gly-Tyr-Met)_2_B] differed in cell culture conditions. This difference could be attributed to the stabilising effect of the TrCys group in comparison with the Met group. TrCys and Met residues are involved in binding to the gold surface. The higher binding of the PBH (Gly-Tyr-TrCys)_2_B to the gold in comparison with the PBH (Gly-Tyr-Met)_2_B is due to the additional aromatic interactions of the TrCys residue. The bulkier group, TrCys, may contribute to protecting individual NPs from assembling into larger agglomerates, thereby leading to the stability of Au[(Gly-Tyr-TrCys)_2_B] agglomerates. In addition, as revealed by elemental analysis, Au[(Gly-Tyr-TrCys)_2_B] was stabilised by 40 PBH units in comparison with 7 PBH units for Au[(Gly-Tyr-Met)_2_B]. Similar considerations can be made for Au[(TrCys)_2_B] and Au[(Met)_2_B]. Au[(TrCys)_2_B] was stable up to 4 h and formed smaller agglomerates over time compared to Au[(Met)_2_B]. The stabilisation of Au[(TrCys)_2_B] was achieved with 97 PBH units compared to 57 units for Au[(Met)_2_B]. It appears that the TrCys group also conferred stability upon Au[(TrCys)_2_B]. Overall, these findings suggest that the TrCys residue and the steric bulk of PBH (Gly-Tyr-TrCys)_2_B are responsible for the remarkable stability of Au[(Gly-Tyr-TrCys)_2_B] agglomerates.

The observations reported here have a major implication for the use of specific PBH capping agents in nanomaterial science. By applying PBH capping agents with different structures, the physico-chemical properties of AuNPs can be manipulated, thus affording tunability in diverse environments.

Interestingly, we observed that the two PBH-capped AuNPs that showed increased stability, namely Au[(Gly-Tyr-TrCys)_2_B] and Au[(TrCys)_2_B], also produced the highest increase in ROS levels. However, significant ROS production was detected only at the two highest doses (50 and 100 μg/ml), thus indicating the feasibility of use at lower concentrations. Oxidative stress induction has been proposed as the principal mechanism of toxicity for many forms of NPs [57-59], including AuNPs [60]. Although the exact biological mechanism behind the action of the AuNPs was not determined in this study, we reveal that they all have the capacity to produce increased levels of ROS. However, the extent of this production differed depending on the PBH structures attached to the AuNP and the medium environment. ROS levels twofold higher than control levels were recorded after exposure to 100 μg/ml Au[(Gly-Tyr-TrCys)_2_B]. In contrast, exposure to the same concentration of Au[(Gly-Tyr-Met)_2_B] elicited only a slight increase in ROS production, which was 50% higher than control levels. The presence or absence of serum also influenced the oxidative stress response to the PBH-capped AuNPs. Those that caused the highest increase in ROS levels in EMEM/S- had a significantly attenuated capacity to induce ROS in the Hep G2 cells in EMEM/S+ medium. For instance, Au[(Gly-Tyr-TrCys)_2_B] AuNPs elicited the highest levels of ROS in EMEM/S-, and this effect was weakened in EMEM/S+, despite this NP having the same size distribution in both mediums (±10 nm). It could therefore be assumed that the attenuated ROS induction observed for all the NPs in EMEM/S+ is not related to size but specifically to serum coating. Merhi et al. [61] showed that endocytosis decreases when NPs are exposed to increasing concentrations of fetal calf serum and bovine serum albumin.

How the AuNPs interact with the cells or whether the different PBH capping agents influence the capacity of the particles to enter cells were not addressed extensively in this study. However, some observations and remarks can be made on the basis of our results. It is known that differently charged functional groups have different associations with cells. In this study, all zeta potentials were negative due to the presence of carboxylate (COO^−^) groups on the attached peptide-biphenyl coatings. Using silica NPs modified with amine and carboxyl functional groups and the murine macrophage cell line (RAW264.7), Nabeshi et al. [62] showed that while amine-functionalised silica NPs absorbed to the plasma membrane, carboxyl functionalities penetrated deeper intracellularly. This finding would suggest that these carboxyl groups bury themselves inside the cell membrane. Thus, the increased biological activity of Au[(Gly-Tyr-TrCys)_2_B] may be explained not only by its stability, remaining in individual AuNP agglomerates of approximately 200 nm in size but also by the presence of free carboxyl groups interacting with cellular components. In addition, studies show that the aromatic structures of tyrosine residues are important regulators of NP cellular uptake (referred to as the aromatic structure hypothesis) [63]. According to these studies, the tyrosine residues in the PBH cap of Au[(Gly-Tyr-TrCys)_2_B] NPs might enhance the cellular uptake. Using Hep G2 cells, Yuan et al. [64] demonstrated that hydroxyapatite NPs as large as 175 nm are taken up by the cells but do not penetrate the nuclear membrane and are confined to the perinuclear region. However, Johnston et al. [65], who also studied the uptake and intracellular fate of NPs in Hep G2 cells, came to the conclusion that the internalisation of 200 nm negatively charged carboxylated polystyrene NPs was limited because of size.

If the PBH-capped AuNPs are taken up, their strong affinity for GSH, together with the significant increase in ROS production, as illustrated in this study, would suggest that the AuNPs act on the same mechanism of oxidative stress as that proposed by Gao et al. [66]. These authors hypothesised that AuNP-induced oxidative stress in the HL7702 human liver cell line is related to the binding of these NPs to endogenous antioxidants (GSH), leading to complete depletion after 48 h. The increase in surface area associated with the decrease in size allows for more GSH binding and thus depletion. They also reported that the extent of oxidative stress depends on NP access to cytosolic GSH or mitochondrial GSH reserves. Hence, increased oxidative stress may occur with smaller NPs. This notion would explain the different levels of ROS production observed in this study, in particular the higher ROS levels elicited by Au[(Gly-Tyr-TrCys)_2_B] (the AuNPs present in the smallest hydrodynamic size, as shown by DLS).

Evidence of dark assemblies in Hep G2 cells exposed to AuNP Au[(Gly-Tyr-TrCys)_2_B] would suggest cellular interaction/internalisation; however, further studies are needed. Cells undergoing autophagy have clearly visible autophagosomes, which form around degraded cellular components. The dark assemblages present in Hep G2 cells after exposure to Au[(Gly-Tyr-TrCys)_2_B] resemble these autophagosomes. Li et al. [67] proposed a cell survival mechanism of autophagy upon exposure to AuNPs. This mechanism has been studied further by Ma et al. [68], who showed that AuNPs that are taken up and accumulate in lysosomes induce autophagosome accumulation through the blockage of the autophagy flux. This observation supports the findings in this study for Au[(Gly-Tyr-TrCys)_2_B]. In this case, despite the high levels of ROS produced, the cells did not succumb to the same loss in viability as that registered for the other NPs at 48 h of exposure. This phenomenon was observed only for cells exposed to the AuNP Au[(Gly-Tyr-TrCys)_2_B], thus suggesting that the unique state of this NP in the culture medium influences the NP-cell interaction.

In fact, AuNPs eliciting the lowest increase in ROS levels after 24 h also showed the greatest loss in viability after 48 h of incubation: exposure to Au[(Gly-Trp-Met)_2_B], Au[(Gly-Tyr-Met)_2_B] and Au[(Met)_2_B] reduced viability to 69%, 71% and 68%, respectively. These AuNPs all formed large agglomerates and had Met groups in their PBH-capping agents.

Several considerations need to be made when studying NP toxicity. One must be aware that NPs may interact unfavourably with assay components. The AuNPs described herein absorb at the same wavelength as those used for the MTT cytotoxicity assay (570 nm) and NRU assay (550 nm). NP interferences with commonly used toxicity assays, such as NRU and MTT, have been reported previously [69,70]. In addition, AuNP interference was also observed when carrying out the GSH/GSSG ratio assay. Care should be taken when interpreting results in order to avoid false positive results. One should also consider that the physico-chemical state of the NP under distinct assay conditions may also lead to differences in levels of interference. All of these factors must not be overlooked.

## Conclusions

Here, we prepared AuNPs using several PBHs as capping agents and studied the influence of the structure of these agents on the physico-chemical state and biocompatibility of the resulting NPs. All the AuNPs tested showed excellent dispersibility in water and form stable agglomerates under culture conditions when serum was present. One PBH-capped AuNP preparation, namely (Au[(Gly-Tyr-TrCys)_2_B]), showed unique physico-chemical properties presenting agglomerates (approximately 200 nm) that remained in the same size distribution under cell culture conditions as when suspended in water, even in the absence of serum. Interestingly, these AuNPs elicited the highest oxidative stress response, with evidence of a unique biological interaction that did not lead to a reduction in Hep G2 cell viability after 48 h of exposure. Our findings suggest that these particular PBH-capped AuNPs exerts a distinct effect on the Hep G2 cell line that is governed by their particular conformation, which is controlled by the chemical structure of their capping agent (Gly-Tyr-TrCys)_2_B. Given the distinct cellular morphology after exposure to these AuNPs and previous reports of AuNP mechanisms of interactions with biological systems, we propose that the Hep G2 cells undergo a cell survival mechanism of autophagy upon exposure to these AuNPs, thus supporting the notion of a cellular interaction/internalisation of these AuNPs. Given the relevance of interaction/internalisation, further research efforts should address the applicability of these AuNPs in drug delivery systems.

## Competing interests

The authors declare that they have no competing interests.

## Authors’ contributions

YP, BH and EM were all involved in the chemical synthesis and design of the peptide-biphenyl hybrid-capped AuNPs. YP and MC performed the physico-chemical characterization of the AuNPs. All toxicity studies were validated and performed by MC and supervised and coordinated by MLFC. MLFC and JMN were involved in the conceptual design of toxicity experiments, data analysis and interpretation and assisted in the preparation of the manuscript. MC and YP drafted the manuscript and figures. All authors read and approved the final manuscript.

## Supplementary Material

Additional file 1 Synthesis of PBHs. Click here for file

Additional file 2: Figure S1 1H NMR spectrum of free PBH (Met)2B (top) in DMSO-d6 and 1H NMR spectrum of AuNP Au[(Met)2B] (bottom) in D2O. Click here for file

Additional file 3: Figure S2 UV–vis absorption spectra of AuNPs **(a)** Au[(Gly-Tyr-Met)2B], (**b**) Au[(Gly-Tyr-TrCys)2B], **(c)** Au[(Gly-Trp-Met)2B], **(d)** Au[(Met)2B] and **(e)** Au[(TrCys)2B], in water and EMEM/+, each at a concentration of 100 μg/ml and a different time 0, 2, 4 and 24 h after incubation at 37°C. Click here for file
