# Supplementary material for: Peptide-biphenyl hybrid-capped AuNPs: stability and biocompatibility under cell culture conditions
Source: Nanoscale Res Lett. 2013 Jul 6;8(1):315. doi: 10.1186/1556-276X-8-315 (PMC3716793; doi:10.1186/1556-276X-8-315)
Supplement: Additional file 1 — Synthesis of PBHs. [file 1556-276X-8-315-S1.pdf]

Additional file 1.

1. Synthesis of PBHs

1.1. General Procedure for Peptide Coupling (GP 1): HOBt (2.6 mmol), Et_3N (3.0 mmol), DCC (2.6 mmol), and DMAP (0.1 mmol) were sequentially added to a stirred solution of the biphenyl dicarboxylic acid compound (1.0 mmol) and the HCl salt of the corresponding amino ester compound (2.6 mmol) in DMF (0.2 M) at room temperature. Stirring was continued for 10-18 h (TLC control). Solvent was evaporated *in vacuo,* and the residue was dissolved in AcOEt and washed with 5% aqueous HCl, saturated $NaHCO_3$ solution, and brine. The organic layer was dried ($MgSO_4$) and concentrated. Purification was accomplished by chromatography with AcOEt/hexane mixture as eluent.

1.2. General Procedure for the Hydrolysis of Methyl Esters (GP 2): LiOH (2.0 equivalent per ester functionality) was portion wise added to a solution of the methyl ester in 1:1 (v/v) THF/H_2O (0.1 M). The mixture was stirred at room temperature for 2-6 h (TLC control). THF was evaporated, and then the aqueous phase was acidified with 5% aqueous HCl until pH 1-2 and extracted with AcOEt. The organic layer was then washed with brine, dried ($MgSO_4$), and concentrated *in vacuo* to give the pure acid.

Flash column chromatography was performed using silica gel (60-Å pore size, 40–63 µm, Merck). Reactions were followed using thin-layer chromatography (TLC) on silica gel-coated plates (Merck 60 F254). Detection was performed with UV light and/or by charring at ca. 150 °C after dipping into an aqueous solution of potassium permanganate ($KMnO_4$), or an ethanolic solution of phosphomolybdic acid (PMA), or an ethanolic solution of ninhydrin. Yields refer to chromatographically and spectroscopically (^{1}H-NMR) homogeneous material, unless otherwise stated.

1.3. Synthesis of PBH ***(MetOMe)₂B****.* A solution of methionine methyl ester hydrochloride (15.0 g, 75.7 mmol) and Et_3N (20.9 ml, 151.4 mmol) in CH_2Cl_2 (100 mL) was added dropwise to a soln. of 1, 1'-biphenyl-2, 2'-dicarbonyl dichloride [1] (9.6 g, 34.4 mmol) in CH_2Cl_2 (200 mL) at 0°C. The mixture was allowed to warm to r.t., and stirring was continued for 15 h (TLC control). Solvent was evaporated, and the residue was dissolved in AcOEt and washed with 5% aq. HCl, sat. aq. $NaHCO_3$ soln. and brine. The organic layer was dried ($MgSO_4$) and concentrated. Purification was accomplished by chromatography with AcOEt/hexane (3:7) mixtures to afford **$(MetOMe)_2B$** (15.37 g, 84%) as a white solid. M.p.: 51-52 ^oC. ^{1}H-NMR (300 MHz, DMSO-*d_6*) δ (ppm): 8.89 (d, 2H, *J* = 7.8 Hz, NH), 7.46 (m, 6H, H_{Ar}), 7.03 (m, 2H, H_{Ar}), 4.31 (m, 2H, C<u>H</u>CH_2), 3.33 (s, 6H, OCH_3), 2.13 (m, 4H, C<u>H_2</u>), 1.94 (s, 6H, SC<u>H_3</u>), 1.80-1.64 (m, 4H, C<u>H_2</u>). ^{13}C-NMR (75 Mz, DMSO-*d_6*) δ (ppm): 172.3 (2C), 170.4 (2C), 139.3 (2C), 136.1 (2C), 130.1 (4C) 128.2 (4C), 52.6 (2C), 51.9 (2C), 31.0 (2C), 29.9 (2C), 15.1 (2C). HRMS-ESI (*m/z*): Calc. for $[M+H]^+$= 533.1774, found: 533.1771.

1.4. Synthesis of ***<u>(Met)₂B</u>****.* From **$(MetOMe)_2B$**, according to GP 2, **$(Met)_2B$** (92% yield) was obtained as a white solid. M.p.**:** 88-89 ^oC. ^{1}H-NMR (300 MHz, DMSO-d_6) δ (ppm): 12.60 (broad s, 2H, COOH), 8.73 (d, 2H, J = 8.1 Hz, NH), 7.44 (m, 6H, H_{Ar}), 7.04 (m, 2H_{Ar}), 4.22 (m, 2H, C<u>H</u>CH_2), 2.11 (m, 4H, C<u>H_2</u>), 1.95 (s, 6H, SC<u>H_3</u>), 1.77 (m, 2H, C<u>H</u>H), 1.64 (m, 2H, CH<u>H</u>). ^{13}C-NMR (75 Mz, DMSO-d_6) δ (ppm): 173.4 (2C), 169.9 (2C), 139.4 (2C), 136.4 (2C), 130.1 (4C) 128.2 (4C), 51.9 (2C), 31.2 (2C), 30.0 (2C), 15.1 (2C). FTIR ν_{max} (cm^{-1}): 3217, 3059, 2915, 1719, 1633, 1538, 1437,

1267, 1222, 1174, 1089, 1005, 956, 872, 756, 716, 654. HRMS-ESI (*m/z*): Calc. for $[M+H]^+$= 505.1461, found: 505.1455.

1.5. Synthesis of PBH ***(TyrOMe-Met)$_2$B***. From **(Met)$_2$B**, according to GP 1, **(TyrOMe-Met)$_2$B** (61% yield) was obtained as a white solid. M.p. 100-102 ^{o}C. ^{1}H-NMR (300 MHz, DMSO-d_6, 50°C) δ (ppm)**:** 9.24 (s, 2H, OH), 8.47 (d, 2H, J = 8.4 Hz, NH), 8.17 (broad s, 2H, NH), 7.50 – 7.25 (m, 6H, H_{Ar}), 7.00 (m, 2H, H_{Ar}), 6.93 (d, 4H, J = 8.4 Hz, H_{Ar}), 6.64 (d, 4H, J = 8.4 Hz, H_{Ar}), 4.29 (m, 4H, CHCOOCH$_3$), 3.53 (s, 6H, OCH$_3$), 2.78 (m, 4H, CH$_2$), 2.07 (m, 4H, CH$_2$), 1.94 (s, 6H, SCH$_3$), 1.60 (m, 4H, CH$_2$S). ^{13}C-NMR (75 Mz, DMSO-d_6) δ (ppm): 172.5 (2C), 171.3 (2C), 169.4 (2C), 156.7 (2C), 139.4 (2C), 136.4 (2C), 130.6 (4C), 129.9 (4C) 128.0 (4C), 127.4 (2C), 115.7 (4C), 54.6 (2C), 52.6 (2C), 52.4 (2C), 36.5 (2C), 32.6 (2C), 29.8 (2C), 15.2 (2C). HRMS-ESI (*m/z*): Calc. for $[M+H]^+$= 859.3041 , found: 859.3036.

1.6. Synthesis of PBH ***(Tyr-Met)$_2$B***. From **(TyrOMe-Met)$_2$B**, according to GP 2, **(Tyr-Met)$_2$B** (89% yield) was obtained as a white solid. M.p. 127-129°C. ^{1}H-NMR (300 MHz, DMSO-d_6) δ (ppm): 9.21 (s, 2H, OH), 8.46 (d, 2H, J = 8.4 Hz, NH), 7.94 (broad s, 2H, NH), 7.50 – 7.25 (m, 6H, H_{Ar}), 7.00 (m, 2H, H_{Ar}), 6.95 (d, 4H, J = 8.4 Hz, H_{Ar}), 6.64 (d, 4H, J = 8.4 Hz, H_{Ar}), 4.27 (m, 4H, CHCOOCH$_3$), 2.79 (m, 4H, CH$_2$), 2.07 (m, 4H, CH$_2$), 1.93 (s, 6H, SCH$_3$), 1.60 (m, 4H, CH$_2$S) ^{13}C-NMR (75 Mz, DMSO-d_6) δ (ppm): 173.4 (2C), 171.1 (2C), 169.4 (2C), 156.7 (2C), 139.4 (2C), 136.5 (2C), 130.6 (4C), 129.9 (4C) 128.0 (4C), 127.8 (2C), 115.7 (4C), 54.3 (2C), 52.8 (2C), 36.6 (2C), 32.6 (2C), 29.8 (2C), 15.2 (2C). HRMS-ESI (*m/z*): Calc. for $[M+H]^+$= 831.2728, found: 831.2726.

1.7. Synthesis of PBH ***(GlyOMe-Tyr-Met)$_2$B***. From **(Tyr-Met)$_2$B**, according to GP 1, **(GlyOMe-Tyr-Met)$_2$B** (59% yield) was obtained as a white solid. M.p. 108-110 ^{o}C. ^{1}H-NMR (300 MHz, DMSO-d_6) δ (ppm): 9.15 (s, 2H, OH), 8.45 (m, 4H, NH), 7.79 (broad s, 2H, NH), 7.78 (broad s, 2H, NH), 7.39 (m, 6H, H_{Ar}), 7.01 (m, 2H, H_{Ar}), 6.97 (d, 4H, J = 8.2 Hz, H_{Ar}), 6.62 (d, 4H, J = 8.2 Hz, H_{Ar}), 4.42 (m, 2H, CH), 4.19 (m, 2H, CH), 3.83 (m, 4H, CH$_2$), 3. 61 (s, 6H, OCH$_3$), 2.84 (m, 2H, CHH), 2.63 (m, 2H, CHH), 2.01 (m, 4H, CH$_2$), 1.92 (s, 6H), 1.53 (m, 4H, CH$_2$S). ^{13}C-NMR (75 Mz, DMSO-d_6) δ (ppm): 171.5 (2C), 171.5 (2C), 170.1 (2C), 168.9 (2C), 155.8 (2C), 138.74 (2C), 135.8 (2C), 130.1 (4C), 129.4 (4C) 127.4 (6C), 114.8 (4C), 53.7 (2C), 52.5 (2C), 51.7 (2C), 40.3 (2C), 36.8 (2C), 31.9 (2C), 29.2 (2C), 14.5 (2C). HRMS-ESI (*m/z*): Calc. for $[M+H]^+$= 973.347, found: 973.3484.

1.8. Synthesis of ***(Gly-Tyr-Met)$_2$B***. From **(GlyOMe-Tyr-Met)$_2$B**, according to GP 2, **(Gly-Tyr-Met)$_2$B** (77% yield) was obtained as a white solid. M.p. 137-139 ^{o}C. ^{1}H-NMR (300 MHz, DMSO-d_6) δ (ppm): 8.50 (d, 2H, J = 8.0 Hz, NH), 8.22 (broad s, 2H, NH), 7.78 (broad s, 2H, NH), 7.38 (m, 6H, H_{Ar}), 7.01 (m, 2H, H_{Ar}), 6.97 (d, 4H, J = 8.4 Hz, H_{Ar}), 6.61 (d, 4H, J = 8.4 Hz, H_{Ar}), 4.42 (m, 2H, CH), 4.17 (m, 2H, CH), 3.71 (m, 4H, CH$_2$), 2.87 (m, 2H, CHH), 2.63 (m, 2H, CHH), 2.00 (m, 4H, CH$_2$), 1.92 (s, 6H), 1.52 (m, 4H, CH$_2$S). ^{13}C-NMR (75 Mz, DMSO-d_6) δ (ppm): 171.3 (2C), 171.1 (2C), 170.2 (2C), 169.9 (2C), 155.8 (2C), 138.7 (2C), 135.8 (2C), 130.2 (4C), 129.4 (4C) 127.6 (6C), 114.8 (4C), 53.8 (2C), 52.6 (2C), 41.1 (2C), 37.0 (2C), 32.9 (2C), 29.2 (2C), 14.5 (2C). FTIR ν_{max} (cm^{-1}): 3286, 1726, 1634, 1514, 1439, 1224, 1113, 830, 759, 649. HRMS-ESI (*m/z*): Calc. for $[M+H]^+$= 945.0675, found: 945.0670.

1.9. Synthesis of PBH ***(TrpOMe-Met)$_2$B***. From **(Met)$_2$B**, according to GP 1, **(TrpOMe-Met)$_2$B** (66% yield) was obtained as a white solid. M.p. 87-89 ^{o}C. ^{1}H-NMR (300 MHz, DMSO-d_6, 50°C) δ (ppm): 10.85 (s, 2H, NH), 8.63 (broad s, 2H, NH), 8.30 (broad d, 2H, J = 6.1 Hz, NH), 7.45 (m, 4H, H_{Ar}), 7.32 (m, 6H, H_{Ar}), 7.02 (m, 8H, H_{Ar}), 4.45 (d, 2H,

C$\underline{H}$), 4.36 (m, 2H, C$\underline{H}$), 3.53 (s, 6H, OC$\underline{H}_3$), 3.06 (m, 4H, C$\underline{H}_2$), 2.11 (m, 4H, C$\underline{H}_2$), 1.95 (s, 6H, SC$\underline{H}_3$), 1.66 (m, 4H, C$\underline{H}_2$S). ^{13}C-NMR (75 Mz, DMSO-d_6) δ (ppm): 172.9 (2C), 171.0 (2C), 169.9 (2C), 138.7 (2C), 136.1 (2C), 135.8 (2C), 129.3 (4C) 127.3 (4C), 127.1 (2C), 123.9 (2C), 120.9 (2C), 118.4 (2C), 117.9 (2C), 111.4 (2C), 108.9 (2C), 53.0 (2C), 52.0 (2C), 51.8 (2C), 31.7 (2C), 29.1 (2C), 26.8 (2C),14.5 (2C). HRMS-ESI (*m/z*): Calc. for $[M+H]^+$= 905.3361, found: 905.3364.

*1.10. Synthesis of PBH **(Trp-Met)$_2$B**.* From **(TrpOMe-Met)$_2$B**, according to GP 2, **(Trp-Met)$_2$B** (88% yield) was obtained as a white solid. M.p. 122-124 ^{o}C. ^{1}H-NMR (300 MHz, DMSO-d_6, 30°C) δ (ppm): 10.85 (s, 2H, NH), 8.63 (broad s, 2H, NH), 8.30 (broad s, 2H, N$\underline{H}$), 7.45 (m, 4H, H_{Ar}), 7.32 (m, 6H, H_{Ar}), 7.02 (m, 8H, H_{Ar}), 4.45 (d, 2H, C$\underline{H}$), 4.36 (m, 24H, C$\underline{H}$), 3.53 (s, 6H, OC$\underline{H}_3$), 3.06 (m, 4H, C$\underline{H}_2$), 2.11 (m, 4H, C$\underline{H}_2$), 1.95 (s, 6H, SC$\underline{H}_3$), 1.66 (m, 4H, C$\underline{H}_2$S). ^{13}C-NMR (75 Mz, DMSO-d_6) δ (ppm): 173.5 (2C), 171.2 (2C), 169.4 (2C), 139.1 (2C), 136.4 (2C), 136.2 (2C), 129.7 (2C), 129.5 (2C), 127.7 (4C), 127.6 (2C), 124.2 (2C), 121.2 (2C), 118.7 (2C), 118.5 (2C), 111.7 (2C), 109.7 (2C), 53.3 (2C), 52.6 (2C), 32.2 (2C), 29.5 (2C), 27.2 (2C),14.9 (2C). HRMS-ESI (*m/z*): Calc. for $[M+H]^+$= 877.3048, found: 877.3051.

*1.11. Synthesis of **(GlyOMe-Trp-Met)$_2$B**.* From **(Trp-Met)$_2$B**, according to GP 1, **(GlyOMe-Trp-Met)$_2$B** (59% yield) was obtained as a white solid. M.p. 107-109°C. ^{1}H-NMR (300 MHz, DMSO-d_6, 50°C) δ (ppm): 10.80 (s, 2H, N$\underline{H}$), 8.59 (broad s, 2H, NH), 8.46 (broad s, 2H, N$\underline{H}$), 7.96 (d, J= 7.3 Hz, 2H, N$\underline{H}$), 7.55 (m, 2H, H_{Ar}), 7.42 (m, 2H, H_{Ar}), 7.32 (m, 6H, H_{Ar}), 7.03 (m, 8H, H_{Ar}), 4.53 (m, 2H, C$\underline{H}$), 4.26 (m, 2H, C$\underline{H}$), 3.83 (m, 4H, C$\underline{H}_2$), 3.61 (s, 6H, OC$\underline{H}_3$), 3.08 (m, 2H, C$\underline{H}$H), 2.93 (m, 2H, CH$\underline{H}$), 2.11 (m, 4H, C$\underline{H}_2$), 1.93 (s, 6H, SC$\underline{H}_3$), 1.60 (m, 4H, C$\underline{H}_2$S). ^{13}C-NMR (75 Mz, DMSO-d_6) δ (ppm): 171.8 (2C), 170.6 (2C), 170.1 (2C), 169.1 (2C), 138.7 (2C), 136.0 (2C), 135.8 (2C), 129.3 (4C) 127.3 (6C), 123.8 (2C), 120.8 (2C), 118.3 (2C), 118.2 (2C), 111.3 (2C), 109.5 (2C), 53.0 (2C), 52.4 (2C), 51.7 (2C), 40.7 (2C), 31.7 (2C), 29.2 (2C), 27.8 (2C),14.5 (2C). HRMS-ESI (*m/z*): Calc. for $[M+H]^+$= 1019.379, found: 1019.375.

*1.12. Synthesis of **(Gly-Trp-Met)$_2$B**.* From **(GlyOMe-Trp-Met)$_2$B**, according to GP 2, **(Gly-Trp-Met)$_2$B** (88% yield) was obtained as a white solid. M.p 130-132°C. ^{1}H-NMR (300 MHz, DMSO-d_6, 50°C) δ (ppm): 10.78 (s, 2H, NH), 8.59 (broad s, 2H, NH), 8.28 (broad s, 2H, NH), 7.94 (d, J= 7.3 Hz, 2H, NH), 7.55 (m, 2H, H_{Ar}), 7.42 (m, 2H, H_{Ar}), 7.32 (m, 6H, H_{Ar}), 7.03 (m, 8H, H_{Ar}), 4.54 (m, 2H, CH), 4.26 (m, 2H, CH), 3.73 (m, 4H, CH_2), 3.10 (m, 2H, CHH), 2.93 (m, 2H, CHH), 2.11 (m, 4H, CH_2), 1.93 (s, 6H, SCH_3), 1.60 (m, 4H, CH_2S). ^{13}C-NMR (75 Mz, DMSO-d_6) δ (ppm): 171.5 (2C), 171.2 (2C), 170.6 (2C), 169.1 (2C), 138.7 (2C), 136.0 (4C), 129.4 (4C) 127.4 (6C), 123.8 (2C), 120.8 (2C), 118.4 (2C), 118.2 (2C), 111.3 (2C), 109.5 (2C), 53.0 (2C), 52.5 (2C), 40.9 (2C), 31.7 (2C), 29.3 (2C), 27.8 (2C),14.5 (2C). FTIR ν_{max} (cm^{-1}): 3286, 2918, 1718, 1634, 1523, 1435, 1341, 1222, 1098, 1011, 872, 742, 652. HRMS-ESI (*m/z*): Calc. for $[M+H]^+$= 991.3477, found: 961.3466.

---

[1] Ugrinova V, Noll BC, Brown SN: **Mononucleating Bis(β-diketonate) Ligands and Their Titanium(IV) Complexes.** *Inorg Chem* 2006, **45**:10309-20.
