# Supplementary material for: Peptide-biphenyl hybrid-capped AuNPs: stability and biocompatibility under cell culture conditions
Source: Nanoscale Res Lett. 2013 Jul 6;8(1):315. doi: 10.1186/1556-276X-8-315 (PMC3716793; doi:10.1186/1556-276X-8-315)
Supplement: Additional file 2: Figure S1 — 1H NMR spectrum of free PBH (Met)2B (top) in DMSO-d6 and 1H NMR spectrum of AuNP Au[(Met)2B] (bottom) in D2O. [file 1556-276X-8-315-S2.pdf]

Additional file 2.

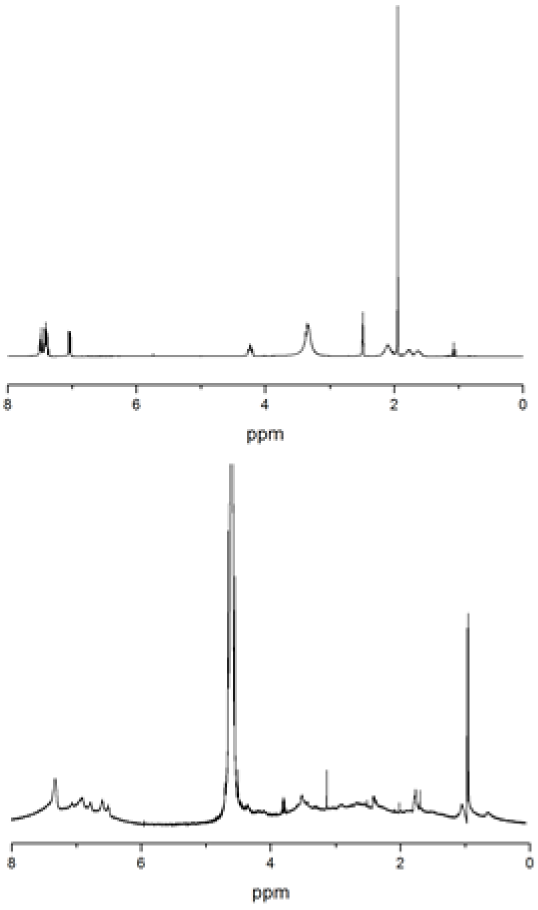

Fig. S1. ^{1}H NMR spectrum of free PBH $(Met)_2B$ (top) in DMSO-d6 and ^{1}H NMR spectrum of AuNP $Au[(Met)_2B]$ (bottom) in D_2O.
