# Supplementary material for: Peptide-biphenyl hybrid-capped AuNPs: stability and biocompatibility under cell culture conditions
Source: Nanoscale Res Lett. 2013 Jul 6;8(1):315. doi: 10.1186/1556-276X-8-315 (PMC3716793; doi:10.1186/1556-276X-8-315)
Supplement: Additional file 3: Figure S2 — UV–vis absorption spectra of AuNPs (a) Au[(Gly-Tyr-Met)2B], (b) Au[(Gly-Tyr-TrCys)2B], (c) Au[(Gly-Trp-Met)2B], (d) Au[(Met)2B] and (e) Au[(TrCys)2B], in water and EMEM/+, each at a concentration of 100 μg/ml and a different time 0, 2, 4 and 24 h after incubation at 37°C. [file 1556-276X-8-315-S3.pdf]

Additional file 3.

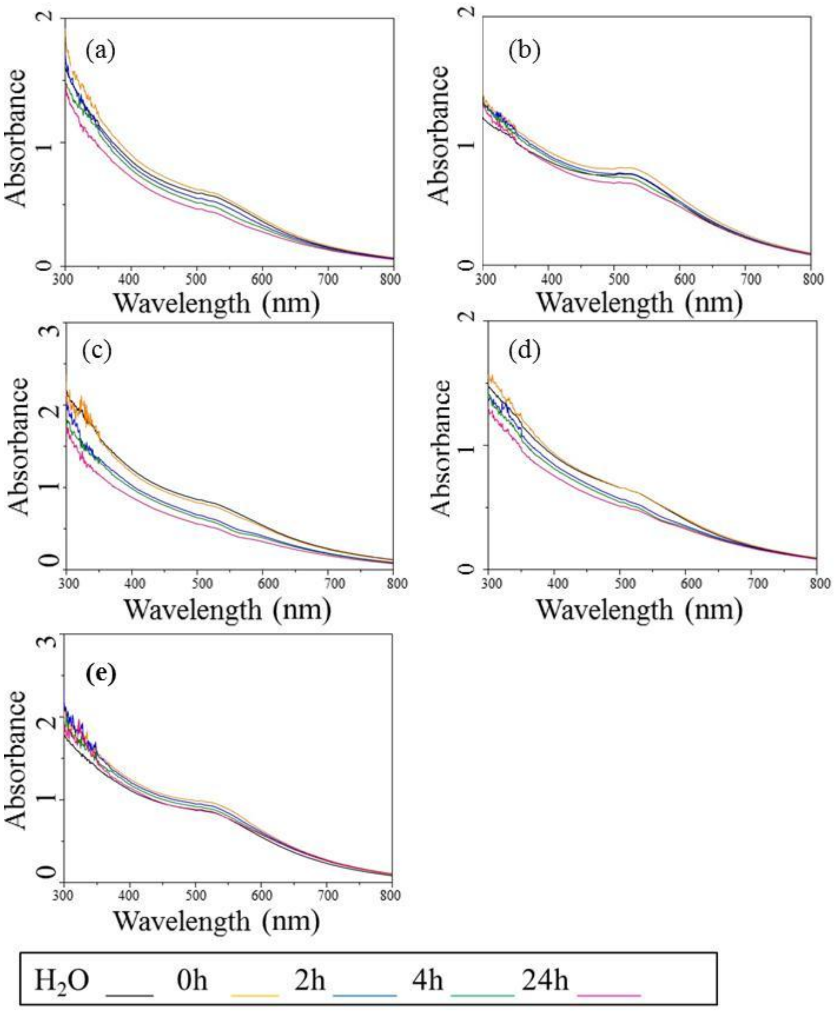

Fig. S2. UV-Vis absorption spectra of AuNPs a) **Au[(Gly-Tyr-Met)$_2$B]**, b) **Au[(Gly-Tyr-TrCys)$_2$B]**, c) **Au[(Gly-Trp-Met)$_2$B]**, d) **Au[(Met)$_2$B]** and e) **Au[(TrCys)$_2$B]**, in water and EMEM/+, each at a concentration of 100 µg/mL and a different time 0, 2, 4, and 24 h after incubation at 37˚C.
